# Expected Logarithm and Negative Integer Moments of a Noncentral *χ*^2^-Distributed Random Variable †

**DOI:** 10.3390/e22091048

**Published:** 2020-09-19

**Authors:** Stefan M. Moser

**Affiliations:** 1 Signal and Information Processing Lab, ETH Zürich, 8092 Zürich, Switzerland; moser@isi.ee.ethz.ch; Tel.: +41-44-632-3624; 2 Institute of Communications Engineering, National Chiao Tung University, Hsinchu 30010, Taiwan

**Keywords:** central *χ*^2^ distribution, chi-square distribution, expected logarithm, exponential distribution, negative integer moments, noncentral *χ*^2^ distribution, squared Rayleigh distribution, squared Rice distribution

## Abstract

Closed-form expressions for the expected logarithm and for arbitrary negative integer moments of a noncentral $\chi^{2}$-distributed random variable are presented in the cases of both even and odd degrees of freedom. Moreover, some basic properties of these expectations are derived and tight upper and lower bounds on them are proposed.

## 1. Introduction

The noncentral $\chi^{2}$ distribution is a family of probability distributions of wide interest. They appear in situations where one or several independent Gaussian random variables (RVs) of equal variance (but potentially different means) are squared and summed together. The noncentral $\chi^{2}$ distribution contains as special cases among others the central $\chi^{2}$ distribution, the exponential distribution (which is equivalent to a squared Rayleigh distribution), and the squared Rice distribution.

In this paper, we present closed-form expressions for the expected logarithm and for arbitrary negative integer moments of a noncentral $\chi^{2}$-distributed RV with even or odd degrees of freedom. Note that while the probability density function (PDF), the moment-generating function (MGF), and the moments of a noncentral $\chi^{2}$-distributed RV are well-known, the expected logarithm and the negative integer moments have only been derived relatively recently for even degrees of freedom [1,2,3,4,5,6], but—to the best of our knowledge—for odd degrees of freedom they have been completely unknown so far. These expectations have many interesting applications. So, for example, in the field of information theory, there is a close relationship between the expected logarithm and entropy, and thus the expected logarithm of a noncentral $\chi^{2}$-distributed RV plays an important role, e.g., in the description of the capacity of multiple-input, multiple-output noncoherent fading channels [1,2]. Many more examples in the field of information theory can be found in [7].

We will see that the expected logarithm and the negative integer moments can be expressed using two families of functions $g_{m}\left(\cdot \right)$ and $h_{n}\left(\cdot \right)$ that will be defined in Section 3. Not unexpectedly, $g_{m}\left(\cdot \right)$ and $h_{n}\left(\cdot \right)$ are not elementary, but contain special functions like the exponential integral function ([8], Sec. 8.21), the imaginary error function [9], or a generalized hypergeometric function ([8], Sec. 9.14). While numerically this does not pose any problem as the required special functions are commonly implemented in many mathematical programming environments, working with them analytically can be cumbersome. We thus investigate $g_{m}\left(\cdot \right)$ and $h_{n}\left(\cdot \right)$ more in detail, present important properties, and derive tight elementary upper and lower bounds to them.

The structure of this paper is as follows. After a few comments about our notation, we will formally define the noncentral $\chi^{2}$ distribution in the following Section 2 and also state some fundamental properties of the expected logarithm and the negative integer moments. In Section 3 we present the two families of functions $g_{m}\left(\cdot \right)$ and $h_{n}\left(\cdot \right)$ that are needed for our main results in Section 4. Section 5 summarizes properties of $g_{m}\left(\cdot \right)$ and $h_{n}\left(\cdot \right)$, and Section 6 presents tight upper and lower bounds on them. Many proofs are deferred to the appendices.

We use upper-case letters to denote random quantities, e.g., *U*, and the corresponding lower-case letter for their realization, e.g., *u*. The expectation operator is denoted by $E\left[\cdot \right]$; ln(·) is the natural logarithm; Γ(·) denotes the Gamma function ([8], Sec. 8.31–8.33); and i is the imaginary number $i≜\sqrt{-1}$. We use $N^{\mathrm{even}}$ to denote the set of all even natural numbers:

(1) $$\begin{array}{c} N^{\mathrm{even}}≜\left\{2,4,6,8,\ldots \right\}. \end{array}$$

Accordingly, $N^{\mathrm{odd}}≜N\backslash N^{\mathrm{even}}$ is the set of all odd natural numbers.

For a function ξ↦f(ξ), $f^{\left(\ell \right)}\left(\xi \right)$ denotes its $\ell^{th}$ derivative:

(2) $$\begin{array}{c} f^{\left(\ell \right)}\left(\xi \right)≜\frac{\;d^{\ell }f\left(\xi \right)}{\;d\xi^{\ell }}. \end{array}$$

The real Gaussian distribution of mean μ∈R and variance $\sigma^{2}>0$ is denoted by $N_{R}\left(\mu ,\sigma^{2}\right)$, while $N_{C}\left(\eta ,\sigma^{2}\right)$ describes the complex Gaussian distribution of mean η∈C and variance $\sigma^{2}>0$. Thus, if $X_{1}$ and $X_{2}$ are independent standard Gaussian RVs, $X_{1},X_{2}∼N_{R}\left(0,1\right)$, $X_{1}⊥\;⊥X_{2}$, then

(3) $$\begin{array}{c} Z≜\frac{1}{\sqrt{2}}X_{1}+i\frac{1}{\sqrt{2}}X_{2} \end{array}$$

is circularly symmetric complex Gaussian, $Z∼N_{C}\left(0,1\right)$.

## 2. The Noncentral $\chi^{2}$ Distribution

**Definition** **1.** *For some n∈N, let ${\left\{X_{k}\right\}}_{k=1}^{n}$ be independent and identically distributed (IID) real, standard Gaussian RVs, $X_{k}∼N_{R}\left(0,1\right)$, let ${\left\{\mu_{k}\right\}}_{k=1}^{n}\in R$ be real constants, and define*

(4) $$\begin{array}{c} \tau ≜\sum_{k=1}^{n}\mu_{k}^{2}. \end{array}$$

*Then the nonnegative RV*

(5) $$\begin{array}{c} U≜\sum_{k=1}^{n}{\left(X_{k}+\mu_{k}\right)}^{2} \end{array}$$

*is said to have a* noncentral $\chi^{2}$ distribution with *n* degrees of freedom and noncentrality parameter τ*. Note that the distribution of U depends on the constants $\left\{\mu_{k}\right\}$ only via the sum of their squares *(4)*. The corresponding PDF is ([10], Ch. 29)*

(6) $$\begin{array}{c} p_{U}\left(u\right)=\frac{1}{2}{\left(\frac{u}{\tau }\right)}^{\frac{n-2}{4}}\;e^{-\frac{\tau +u}{2}}I_{n/2-1}\left(\sqrt{\tau u}\right),\;u\geq 0, \end{array}$$

*where $I_{\nu }\left(\cdot \right)$ denotes the modified Bessel function of the first kind of order ν∈R ([8], Eq. 8.445):*

(7) $$\begin{array}{c} I_{\nu }\left(x\right)≜\sum_{k=0}^{\infty }\frac{1}{k!\;\Gamma (\nu +k+1)}{\left(\frac{x}{2}\right)}^{\nu +2k},\;x\geq 0. \end{array}$$

*For τ=0 we obtain the central $\chi^{2}$ distribution, for which the PDF *(6)* simplifies to*

(8) $$\begin{array}{c} p_{U}\left(u\right)=\frac{1}{2^{\frac{n}{2}}\;\Gamma \left(\frac{n}{2}\right)}\;u^{\frac{n}{2}-1}\;e^{-\frac{u}{2}},\;u\geq 0. \end{array}$$

Note that in this work any RV *U* will always be defined as given in (5). Sometimes we will write $U^{\left[n,\tau \right]}$ to clarify the degrees of freedom *n* and the noncentrality parameter τ of *U*.

If the number of degrees of freedom *n* is even (i.e., if n=2m for some natural number *m*), there exists a second, slightly different definition of the noncentral $\chi^{2}$ distribution that is based on *complex* Gaussian random variables.

**Definition** **2.** *For some m∈N, let ${\left\{Z_{j}\right\}}_{j=1}^{m}$ be IID $∼N_{C}\left(0,1\right)$, let ${\left\{\eta_{j}\right\}}_{j=1}^{m}\in C$ be complex constants, and define*

(9) $$\begin{array}{c} \lambda ≜\sum_{j=1}^{m}{\left|\eta_{j}\right|}^{2}. \end{array}$$

*Then the nonnegative RV*

(10) $$\begin{array}{c} V≜\sum_{j=1}^{m}{\left|Z_{j}+\eta_{j}\right|}^{2} \end{array}$$

*is said to have a* noncentral $\chi^{2}$ distribution with 2m degrees of freedom and noncentrality parameter λ*. It has a PDF*

(11) $$\begin{array}{c} p_{V}\left(v\right)={\left(\frac{v}{\lambda }\right)}^{\frac{m-1}{2}}\;e^{-v-\lambda }I_{m-1}\left(2\sqrt{\lambda v}\right),\;v\geq 0, \end{array}$$

*which in the central case of λ=0 simplifies to*

(12) $$\begin{array}{c} p_{V}\left(v\right)=\frac{1}{\Gamma (m)}\;v^{m-1}\;e^{-v},\;v\geq 0. \end{array}$$

Note that in this work any RV *V* will always be defined as given in (10). Sometimes we will write $V^{\left[m,\lambda \right]}$ to clarify the degrees of freedom 2m and the noncentrality parameter λ of *V*.

**Lemma** **1.** *Let $n\in N^{\mathrm{even}}$ be an even natural number and τ≥0 a nonnegative constant. Then*

(13) $$\begin{array}{c} U^{\left[n,\tau \right]}\overset{L}{=}2V^{\left[n/2,\tau /2\right]}, \end{array}$$

*where “$\overset{L}{=}$” denotes equality in probability law.*

**Proof.**  Let ${\left\{X_{k}\right\}}_{k=1}^{n}$ be IID $∼N_{R}\left(0,1\right)$ and ${\left\{\mu_{k}\right\}}_{k=1}^{n}\in R$ as given in Definition 1. Define m≜n/2∈N and for all j∈{1,…,m},

(14) $$\begin{array}{ccc} Z_{j} & ≜ & \frac{1}{\sqrt{2}}X_{2j-1}+i\frac{1}{\sqrt{2}}X_{2j}, \end{array}$$

(15) $$\begin{array}{ccc} \eta_{j} & ≜ & \frac{1}{\sqrt{2}}\mu_{2j-1}+i\frac{1}{\sqrt{2}}\mu_{2j}. \end{array}$$

Then

(16) $$\begin{array}{ccc} \lambda & ≜ & \sum_{j=1}^{m}{\left|\eta_{j}\right|}^{2} \end{array}$$

(17) $$\begin{array}{ccc} & = & \sum_{j=1}^{m}\left(\frac{1}{2}\mu_{2j-1}^{2}+\frac{1}{2}\mu_{2j}^{2}\right) \end{array}$$

(18) $$\begin{array}{ccc} & = & \frac{1}{2}\sum_{k=1}^{n}\mu_{k}^{2}=\frac{1}{2}\tau \end{array}$$

and

(19) $$\begin{array}{ccc} V^{\left[m,\lambda \right]} & = & \sum_{j=1}^{m}{\left|Z_{j}+\eta_{j}\right|}^{2} \end{array}$$

(20) $$\begin{array}{ccc} & = & \sum_{j=1}^{m}{\left|\frac{1}{\sqrt{2}}X_{2j-1}+i\frac{1}{\sqrt{2}}X_{2j}+\frac{1}{\sqrt{2}}\mu_{2j-1}+i\frac{1}{\sqrt{2}}\mu_{2j}\right|}^{2} \end{array}$$

(21) $$\begin{array}{ccc} & = & \frac{1}{2}\sum_{j=1}^{m}\left({\left(X_{2j-1}+\mu_{2j-1}\right)}^{2}+{\left(X_{2j}+\mu_{2j}\right)}^{2}\right) \end{array}$$

(22) $$\begin{array}{ccc} & = & \frac{1}{2}\sum_{k=1}^{n}{\left(X_{k}+\mu_{k}\right)}^{2} \end{array}$$

(23) $$\begin{array}{ccc} & = & \frac{1}{2}U^{\left[n,\tau \right]}. \end{array}$$

 □

**Proposition** **1** (Existence of Negative Integer Moments)**.** *For ℓ∈N, the negative $\ell^{th}$ moment of a noncentral $\chi^{2}$-distributed RV of n∈N degrees of freedom and noncentrality parameter τ≥0,*

(24) $$\begin{array}{c} E\left[{\left(U^{\left[n,\tau \right]}\right)}^{-\ell }\right], \end{array}$$

*is finite if, and only if,*

(25) $$\begin{array}{c} \ell <\frac{n}{2}. \end{array}$$

**Proof.**  See Appendix A.1. □

**Proposition** **2** (Monotonicity in Degrees of Freedom)**.** *The expected logarithm of a noncentral $\chi^{2}$-distributed RV of n∈N degrees of freedom and noncentrality parameter τ≥0,*

(26) $$\begin{array}{c} E\left[\ln\left(U^{\left[n,\tau \right]}\right)\right], \end{array}$$

*is monotonically strictly increasing in n (for fixed τ).*

*Similarly, for any $\ell \in N\cap \left[1,\frac{n}{2}-1\right]$, the negative $\ell^{th}$ moment of $U^{\left[n,\tau \right]}$,*

(27) $$\begin{array}{c} E\left[{\left(U^{\left[n,\tau \right]}\right)}^{-\ell }\right], \end{array}$$

*is monotonically strictly decreasing in n (for fixed τ).*

**Proof.**  See Appendix A.2. □

**Proposition** **3** (Continuity in Noncentrality Parameter)**.** *For a fixed n, the expected logarithm *(26)* is continuous in τ for every finite τ≥0.*

**Proof.**  See Appendix A.3. □

For completeness, we present here the positive integer moments of the noncentral $\chi^{2}$ distribution.

**Proposition** **4** (Positive Integer Moments)**.** *For any ℓ∈N, the positive $\ell^{th}$ moment of $U^{\left[n,\tau \right]}$ is given recursively as*

(28) $$\begin{array}{ccc} E\left[{\left(U^{\left[n,\tau \right]}\right)}^{\ell }\right] & = & 2^{\ell -1}\left(\ell -1\right)!\;\left(n+\ell \tau \right)+\sum_{j=1}^{\ell -1}\frac{\left(\ell -1\right)!\;2^{j-1}}{(\ell -j)!}\cdot \left(n+j\tau \right)\cdot E\left[{\left(U^{\left[n,\tau \right]}\right)}^{\ell -j}\right]. \end{array}$$

*Thus, the first two moments are*

(29) $$\begin{array}{ccc} E\left[U^{\left[n,\tau \right]}\right] & = & n+\tau , \end{array}$$

(30) $$\begin{array}{ccc} E\left[{\left(U^{\left[n,\tau \right]}\right)}^{2}\right] & = & {\left(n+\tau \right)}^{2}+2n+4\tau . \end{array}$$

*The corresponding expressions for $V^{\left[m,\lambda \right]}$ follow directly from Lemma 1.*

**Proof.**  See, e.g., [10]. □

For the special case of the *central* $\chi^{2}$ distribution (i.e., the case when τ=λ=0), it is straightforward to compute the expected logarithm and the negative integer moments by evaluating the corresponding integrals.

**Proposition** **5** (Expected Logarithm and Negative Integer Moments for Central $\chi^{2}$ Distribution)**.** *For any n,m∈N, we have*

(31) $$\begin{array}{ccc} E\left[\ln\left(U^{\left[n,0\right]}\right)\right] & = & \ln\left(2\right)+\psi \left(\frac{n}{2}\right), \end{array}$$

(32) $$\begin{array}{ccc} E\left[\ln\left(V^{\left[m,0\right]}\right)\right] & = & \psi (m), \end{array}$$

*where ψ(·) denotes the digamma function ([8], Sec. 8.36) (see also *(37)* and *(51)* below).*

*Moreover, for any n,m,ℓ∈N,*

(33) $$\begin{array}{ccc} E\left[{\left(U^{\left[n,0\right]}\right)}^{-\ell }\right] & = & \left\{\begin{array}{cc} \frac{2^{-\ell }\;\Gamma \left(\frac{n}{2}-\ell \right)}{\Gamma \left(\frac{n}{2}\right)} & if\;n\geq 2\ell +1,\\ \infty & if\;n\leq 2\ell , \end{array}\right. \end{array}$$

(34) $$\begin{array}{ccc} E\left[{\left(V^{\left[m,0\right]}\right)}^{-\ell }\right] & = & \left\{\begin{array}{cc} \frac{\Gamma (m-\ell )}{\Gamma (m)} & if\;m\geq \ell +1,\\ \infty & if\;m\leq \ell . \end{array}\right. \end{array}$$

**Proof.**  These results follow directly by evaluating the corresponding integrals using the PDF (8) and (12), respectively. See also (A2) in Appendix A.1 and (A46) in Appendix B. □

## 3. Two Families of Functions

### 3.1. The Family of Functions $g_{m}\left(\cdot \right)$

The following family of functions will be essential for the expected logarithm and the negative integer moments of a noncentral $\chi^{2}$-distributed RV of *even* degrees of freedom.

**Definition** **3.** *([1,2]) For an arbitrary m∈N, we define the function $g_{m}:R_{0}^{+}\to R$,*

(35) $$\begin{array}{c} \xi \mapsto g_{m}\left(\xi \right)≜\left\{\begin{array}{cc} \ln\left(\xi \right)-\mathrm{Ei}\left(-\xi \right)+\sum_{j=1}^{m-1}{\left(-1\right)}^{j}\left[\;e^{-\xi }\left(j-1\right)!-\frac{(m-1)!}{j(m-1-j)!}\right]{\left(\frac{1}{\xi }\right)}^{j} & if\;\xi >0,\\ \psi (m) & if\;\xi =0. \end{array}\right. \end{array}$$

*Here, $\mathrm{Ei}\left(\cdot \right)$ denotes the exponential integral function ([8], Sec. 8.21)*

(36) $$\begin{array}{c} \mathrm{Ei}\left(-x\right)≜-\int_{x}^{\infty }\frac{\;e^{-t}}{t}\;dt,\;x>0, \end{array}$$

*and ψ(·) is the digamma function ([8], Sec. 8.36) that for natural values takes the form*

(37) $$\begin{array}{c} \psi \left(m\right)≜-\gamma +\sum_{j=1}^{m-1}\frac{1}{j}, \end{array}$$

*with γ≈0.577 being Euler’s constant.*

Note that in spite of the case distinction in its definition, $g_{m}\left(\xi \right)$ actually is continuous for all ξ≥0. In particular,

(38) $$\begin{array}{ccc} \lim_{\xi ↓0}\left\{\ln\left(\xi \right)-\mathrm{Ei}\left(-\xi \right)+\sum_{j=1}^{m-1}{\left(-1\right)}^{j}\left[\;e^{-\xi }\;\left(j-1\right)!-\frac{(m-1)!}{j(m-1-j)!}\right]{\left(\frac{1}{\xi }\right)}^{j}\right\} & = & \psi (m) \end{array}$$

for all m∈N. This will follow from Proposition 3 once we have shown the connection between $g_{m}\left(\cdot \right)$ and the expected logarithm (see Theorem 1).

Therefore, its first derivative is defined for all ξ≥0 and can be evaluated to

(39) $$\begin{array}{c} g_{m}^{\left(1\right)}\left(\xi \right)=\left\{\begin{array}{cc} \frac{{\left(-1\right)}^{m}\;\left(m-1\right)!}{\xi^{m}}\left(\;e^{-\xi }-\sum_{j=0}^{m-1}\frac{{\left(-1\right)}^{j}}{j!}\xi^{j}\right) & \mathrm{if}\;\xi >0,\\ \frac{1}{m} & \mathrm{if}\;\xi =0. \end{array}\right. \end{array}$$

Using the following expression for the incomplete Gamma function [11]

(40) $$\begin{array}{c} \Gamma \left(m,z\right)=\left(m-1\right)!\;e^{-z}\sum_{j=0}^{m-1}\frac{z^{j}}{j!}, \end{array}$$

the expression (39) can also be rewritten as

(41) $$\begin{array}{c} g_{m}^{\left(1\right)}\left(\xi \right)=\left\{\begin{array}{cc} {\left(-1\right)}^{m}\xi^{-m}\;e^{-\xi }\left(\Gamma (m)-\Gamma (m,-\xi )\right) & \mathrm{if}\;\xi >0,\\ \frac{1}{m} & \mathrm{if}\;\xi =0. \end{array}\right. \end{array}$$

Note that also $g_{m}^{\left(1\right)}\left(\cdot \right)$ is continuous and that in particular

(42) $$\begin{array}{c} \lim_{\xi ↓0}\left\{\frac{{\left(-1\right)}^{m}\;\left(m-1\right)!}{\xi^{m}}\left(\;e^{-\xi }-\sum_{j=0}^{m-1}\frac{{\left(-1\right)}^{j}}{j!}\xi^{j}\right)\right\}=\frac{1}{m}. \end{array}$$

This can be checked directly using the series expansion of the exponential function to write

(43) $$\begin{array}{ccc} g_{m}^{\left(1\right)}\left(\xi \right) & = & \frac{{\left(-1\right)}^{m}\left(m-1\right)!}{\xi^{m}}\sum_{j=m}^{\infty }\frac{{\left(-1\right)}^{j}}{j!}\xi^{j} \end{array}$$

(44) $$\begin{array}{ccc} & = & \left(m-1\right)!\sum_{k=0}^{\infty }\frac{{\left(-1\right)}^{k}}{(k+m)!}\xi^{k} \end{array}$$

and plug ξ=0 in, or it follows from (63a) in Theorem 3, which shows that $g_{m}^{\left(1\right)}\left(\xi \right)$ can be written as a difference of two continuous functions.

**Figure 1:**
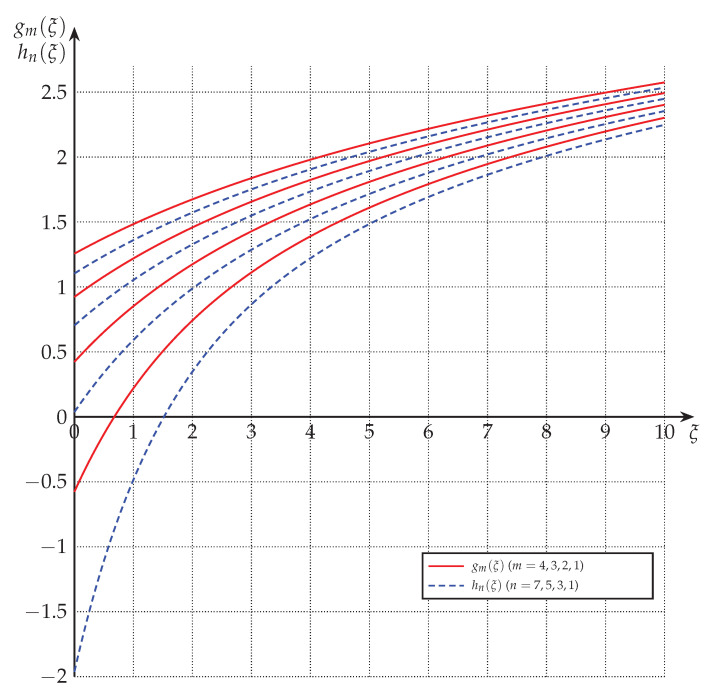
The functions $g_{m}\left(\cdot \right)$ and $h_{n}\left(\cdot \right)$ for m∈{1,2,3,4} and n∈{1,3,5,7}. (Increasing *n* and *m* results in increasing values.)

**Figure 2:**
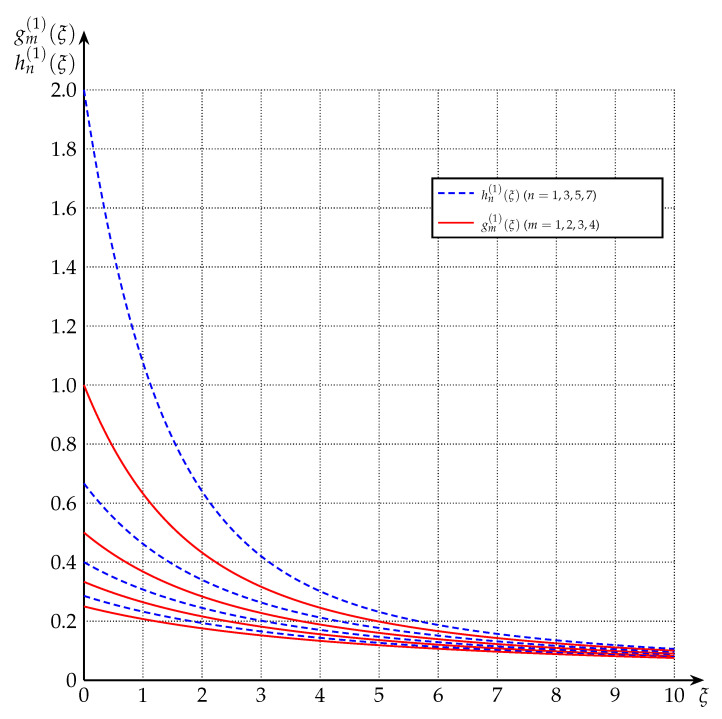
The functions $g_{m}^{\left(1\right)}\left(\cdot \right)$ and $h_{n}^{\left(1\right)}\left(\cdot \right)$ for m∈{1,2,3,4} and n∈{1,3,5,7}. (Increasing *n* or *m* results in decreasing values.)

Figure 1 and Figure 2 depict $g_{m}\left(\cdot \right)$ and $g_{m}^{\left(1\right)}\left(\cdot \right)$, respectively, for various values of *m*.

Note that the $\ell^{th}$ derivative of $g_{m}\left(\cdot \right)$ can be expressed as a finite sum of $g_{m+j}\left(\cdot \right)$ or of $g_{m+j}^{\left(1\right)}\left(\cdot \right)$, see Corollary 2 in Section 5.

### 3.2. The Family of Functions $h_{n}\left(\cdot \right)$

The following family of functions will be essential for the expected logarithm and the negative integer moments of a noncentral $\chi^{2}$-distributed RV of *odd* degrees of freedom.

**Definition** **4.** *For an arbitrary odd $n\in N^{\mathrm{odd}}$, we define the function $h_{n}:R_{0}^{+}\to R$,*

(45) $$\begin{array}{c} \xi \mapsto h_{n}\left(\xi \right)≜\left\{\begin{array}{cc} -\gamma -2\ln\left(2\right)+2\xi \cdot {}_{2}F_{2}\left(1,1;\frac{3}{2},2;-\xi \right)\\ \;+\sum_{j=1}^{\frac{n-1}{2}}{\left(-1\right)}^{j-1}\Gamma \left(j-\frac{1}{2}\right)\cdot \left[\sqrt{\xi }\;e^{-\xi }\mathrm{erfi}\left(\sqrt{\xi }\right)+\sum_{i=1}^{j-1}\frac{{\left(-1\right)}^{i}\;\xi^{i}}{\Gamma \left(i+\frac{1}{2}\right)}\right]{\left(\frac{1}{\xi }\right)}^{j} & if\;\xi >0,\\ \psi \left(\frac{n}{2}\right) & if\;\xi =0. \end{array}\right. \end{array}$$

*Here γ≈0.577 denotes Euler’s constant, ψ(·) is the digamma function ([8], Sec. 8.36), ${}_{2}F_{2}\left(\cdot \right)$ is a generalized hypergeometric function ([8], Sec. 9.14)*

(46) $$\begin{array}{c} {}_{2}F_{2}\left(1,1;\frac{3}{2},2;-\xi \right)≜\frac{\sqrt{\pi }}{2}\sum_{k=0}^{\infty }\frac{{\left(-1\right)}^{k}}{\Gamma \left(\frac{3}{2}+k\right)\;\left(k+1\right)}\;\xi^{k}, \end{array}$$

*and $\mathrm{erfi}\left(\cdot \right)$ denotes the imaginary error function [9]*

(47) $$\begin{array}{c} \mathrm{erfi}\left(\xi \right)≜\frac{2}{\sqrt{\pi }}\int_{0}^{\xi }\;e^{t^{2}}\;dt. \end{array}$$

*Note that one can also use Dawson’s function [12]*

(48) $$\begin{array}{c} D\left(\xi \right)≜\;e^{-\xi^{2}}\int_{0}^{\xi }e^{t^{2}}\;dt \end{array}$$

*to write*

(49) $$\begin{array}{c} \;e^{-\xi }\mathrm{erfi}\left(\sqrt{\xi }\right)=\frac{2}{\sqrt{\pi }}\;D\left(\sqrt{\xi }\right). \end{array}$$

*This often turns out to be numerically more stable.*

Note that $h_{n}\left(\xi \right)$ is continuous for all ξ≥0; in particular,

(50) $$\begin{array}{c} \lim_{\xi ↓0}h_{n}\left(\xi \right)=\psi \left(\frac{n}{2}\right) \end{array}$$

for all $n\in N^{\mathrm{odd}}$. This will follow from Proposition 3 once we have shown the connection between $h_{n}\left(\cdot \right)$ and the expected logarithm (see Theorem 1).

Moreover, note that ([8], Eq. 8.366-3)

(51) $$\begin{array}{c} h_{n}\left(0\right)=\psi \left(\frac{n}{2}\right)=-\gamma -2\ln\left(2\right)+\sum_{j=1}^{\frac{n-1}{2}}\frac{1}{j-\frac{1}{2}}. \end{array}$$

The first derivative of $h_{n}\left(\cdot \right)$ is defined for all ξ≥0 and can be evaluated to

(52) $$\begin{array}{c} h_{n}^{\left(1\right)}\left(\xi \right)=\left\{\begin{array}{cc} \frac{{\left(-1\right)}^{\frac{n-1}{2}}\;\Gamma \left(\frac{n}{2}\right)}{\xi^{\frac{n}{2}}}\left(\;e^{-\xi }\mathrm{erfi}\left(\sqrt{\xi }\right)+\sum_{j=1}^{\frac{n-1}{2}}\frac{{\left(-1\right)}^{j}}{\Gamma \left(j+\frac{1}{2}\right)}\xi^{j-\frac{1}{2}}\right) & \mathrm{if}\;\xi >0,\\ \frac{2}{n} & \mathrm{if}\;\xi =0. \end{array}\right. \end{array}$$

Note that also $h_{n}^{\left(1\right)}\left(\cdot \right)$ is continuous and that in particular

(53) $$\begin{array}{c} \lim_{\xi ↓0}h_{n}^{\left(1\right)}\left(\xi \right)=\frac{2}{n} \end{array}$$

for all $n\in N^{\mathrm{odd}}$. Checking this directly is rather cumbersome. It is easier to deduce this from (76a) in Theorem 5, which shows that $h_{n}^{\left(1\right)}\left(\xi \right)$ can be written as a difference of two continuous functions.

Figure 1 and Figure 2 depict $h_{n}\left(\cdot \right)$ and $h_{n}^{\left(1\right)}\left(\cdot \right)$, respectively, for various values of *n*.

Note that the $\ell^{th}$ derivative of $h_{n}\left(\cdot \right)$ can be expressed as a finite sum of $h_{n+j}\left(\cdot \right)$ or of $h_{n+j}^{\left(1\right)}\left(\cdot \right)$, see Corollary 6 in Section 5.

## 4. Expected Logarithm and Negative Integer Moments

We are now ready for our main results. We will show how the functions $h_{n}\left(\cdot \right)$ and $g_{m}\left(\cdot \right)$ from Section 3 are connected to the expected logarithm and the negative integer moments of noncentral $\chi^{2}$-distributed random variables.

**Theorem** **1** (Expected Logarithm)**.** *For some n∈N and τ≥0, let $U^{\left[n,\tau \right]}$ be as in Definition 1. Then*

(54) $$\begin{array}{c} E\left[\ln\left(U^{\left[n,\tau \right]}\right)\right]=\left\{\begin{array}{cc} \ln\left(2\right)+h_{n}\left(\frac{\tau }{2}\right) & if\;n\in N^{\mathrm{odd}},\\ \ln\left(2\right)+g_{n/2}\left(\frac{\tau }{2}\right) & if\;n\in N^{\mathrm{even}}. \end{array}\right. \end{array}$$

*Similarly, for some m∈N and λ≥0, let $V^{\left[m,\lambda \right]}$ be as in Definition 2. Then*

(55) $$\begin{array}{c} E\left[\ln\left(V^{\left[m,\lambda \right]}\right)\right]=g_{m}\left(\lambda \right). \end{array}$$

**Theorem** **2** (Negative Integer Moments)**.** *For some n∈N and τ≥0, let $U^{\left[n,\tau \right]}$ be as in Definition 1. Then, for any ℓ∈N,*

(56) $$\begin{array}{c} E\left[{\left(U^{\left[n,\tau \right]}\right)}^{-\ell }\right]=\left\{\begin{array}{cc} \frac{{\left(-1\right)}^{\ell -1}}{\left(\ell -1\right)!\;2^{\ell }}\cdot h_{n-2\ell }^{\left(\ell \right)}\left(\frac{\tau }{2}\right) & if\;n\geq 2\ell +1\;\mathrm{and}\;n\in N^{\mathrm{odd}},\\ \frac{{\left(-1\right)}^{\ell -1}}{\left(\ell -1\right)!\;2^{\ell }}\cdot g_{\frac{n}{2}-\ell }^{\left(\ell \right)}\left(\frac{\tau }{2}\right) & if\;n\geq 2\ell +1\;\mathrm{and}\;n\in N^{\mathrm{even}},\\ \infty & \mathrm{if}\;n\leq 2\ell . \end{array}\right. \end{array}$$

*Similarly, for some m∈N and λ≥0, let $V^{\left[m,\lambda \right]}$ be as in Definition 2. Then, for any ℓ∈N,*

(57) $$\begin{array}{c} E\left[{\left(V^{\left[m,\lambda \right]}\right)}^{-\ell }\right]=\left\{\begin{array}{cc} \frac{{\left(-1\right)}^{\ell -1}}{(\ell -1)!}\cdot g_{m-\ell }^{\left(\ell \right)}\left(\lambda \right) & \mathrm{if}\;m\geq \ell +1,\\ \infty & \mathrm{if}\;m\leq \ell . \end{array}\right. \end{array}$$

*In particular, this means that for any n≥3,*

(58) $$\begin{array}{c} E\left[\frac{1}{U^{\left[n,\tau \right]}}\right]=\left\{\begin{array}{cc} \frac{1}{2}\;h_{n-2}^{\left(1\right)}\left(\frac{\tau }{2}\right) & \mathrm{if}\;n\in N^{\mathrm{odd}},\\ \frac{1}{2}\;g_{\frac{n}{2}-1}^{\left(1\right)}\left(\frac{\tau }{2}\right) & \mathrm{if}\;n\in N^{\mathrm{even}}, \end{array}\right. \end{array}$$

*and for any m≥2,*

(59) $$\begin{array}{c} E\left[\frac{1}{V^{\left[m,\lambda \right]}}\right]=g_{m-1}^{\left(1\right)}\left(\lambda \right). \end{array}$$

A proof for these two main theorems can be found in Appendix B.

## 5. Properties

We next investigate the two families of functions $g_{m}\left(\cdot \right)$ and $h_{n}\left(\cdot \right)$ more closely and state some useful properties.

### 5.1. Properties of $g_{m}\left(\cdot \right)$

**Proposition** **6.** For any m∈N, the function $\xi \mapsto g_{m}\left(\xi \right)$ is monotonically strictly increasing and strictly concave ($\xi \in R_{0}^{+}$).

**Proof.**  Using the expression (A104), we have

(60) $$\begin{array}{ccc} g_{m}^{\left(1\right)}\left(\xi \right) & = & \;e^{-\xi }\sum_{k=0}^{\infty }\frac{\xi^{k}}{k!}\;\frac{1}{k+m}, \end{array}$$

(61) $$\begin{array}{ccc} g_{m}^{\left(2\right)}\left(\xi \right) & = & -\;e^{-\xi }\sum_{k=0}^{\infty }\frac{\xi^{k}}{k!}\;\frac{1}{\left(k+m)(k+m+1\right)}, \end{array}$$

i.e., the first derivative of $g_{m}\left(\cdot \right)$ is positive and the second derivative is negative. □

**Proposition** **7.** For any ξ≥0, the function $m\mapsto g_{m}\left(\xi \right)$ is monotonically strictly increasing (m∈N).

**Proof.**  This follows directly from Theorem 1 and Proposition 2. □

**Proposition** **8.** For any m∈N, the function $\xi \mapsto g_{m}^{\left(1\right)}\left(\xi \right)$ is positive, monotonically strictly decreasing, and strictly convex ($\xi \in R_{0}^{+}$).

**Proof.**  The positivity and the monotonicity follow directly from (60) and (61). To see the convexity, use (A104) to write

(62) $$\begin{array}{c} g_{m}^{\left(3\right)}\left(\xi \right)=\;e^{-\xi }\sum_{k=0}^{\infty }\frac{\xi^{k}}{k!}\;\frac{2}{\left(k+m)(k+m+1)(k+m+2\right)}, \end{array}$$

which is positive. □

**Proposition** **9.** For any ξ≥0, the function $m\mapsto g_{m}^{\left(1\right)}\left(\xi \right)$ is monotonically strictly decreasing (m∈N).

**Proof.**  This follows directly from Theorem 2 and Proposition 2. □

**Theorem** **3.** *For all m∈N, ξ≥0, and ℓ∈N, we have the following relations:*

(63a) $$\begin{array}{ccc} g_{m+1}\left(\xi \right) & = & g_{m}\left(\xi \right)+g_{m}^{\left(1\right)}\left(\xi \right), \end{array}$$

(63b) $$\begin{array}{ccc} g_{m+1}^{\left(\ell \right)}\left(\xi \right) & = & g_{m}^{\left(\ell \right)}\left(\xi \right)+g_{m}^{\left(\ell +1\right)}\left(\xi \right). \end{array}$$

**Proof.**  See Appendix C.1. □

**Corollary** **1.** *For any m>1,*

(64) $$\begin{array}{c} E\left[\frac{1}{V^{\left[m,\lambda \right]}}\right]=g_{m}\left(\lambda \right)-g_{m-1}\left(\lambda \right). \end{array}$$

**Proof.**  This follows directly from (63a) and (59). □

**Corollary** **2.** *The ℓth derivative $g_{m}^{\left(\ell \right)}\left(\cdot \right)$ can be written with the help of either the first derivative $g_{m}^{\left(1\right)}\left(\cdot \right)$ or of $g_{m}\left(\cdot \right)$ in the following ways:*

(65) gm(ℓ)(ξ)=∑j=0ℓ−1(−1)j+ℓ−1ℓ−1jgm+j(1)(ξ)

(66) =∑j=0ℓ(−1)j+ℓℓjgm+j(ξ).

**Proof.**  Using $g_{m}^{\left(0\right)}\left(\cdot \right)$ as an equivalent expression for $g_{m}\left(\cdot \right)$, we rewrite (63) as

(67) $$\begin{array}{c} g_{m}^{\left(\ell \right)}\left(\xi \right)=g_{m+1}^{\left(\ell -1\right)}\left(\xi \right)-g_{m}^{\left(\ell -1\right)}\left(\xi \right), \end{array}$$

and recursively apply this relation. □

**Corollary** **3.** *For all m∈N and ξ≥0,*

(68) $$\begin{array}{ccc} g_{m}\left(\xi \right) & = & g_{1}\left(\xi \right)+\sum_{j=1}^{m-1}g_{j}^{\left(1\right)}\left(\xi \right) \end{array}$$

(69) $$\begin{array}{ccc} & = & \ln\left(\xi \right)-\mathrm{Ei}\left(-\xi \right)+\sum_{j=1}^{m-1}g_{j}^{\left(1\right)}\left(\xi \right). \end{array}$$

**Proof.**  We recursively apply (63a) to obtain the relation

(70) $$\begin{array}{ccc} g_{m}\left(\xi \right) & = & g_{m-1}\left(\xi \right)+g_{m-1}^{\left(1\right)}\left(\xi \right) \end{array}$$

(71) $$\begin{array}{ccc} & = & g_{m-2}\left(\xi \right)+g_{m-2}^{\left(1\right)}\left(\xi \right)+g_{m-1}^{\left(1\right)}\left(\xi \right)\\ & \vdots & \end{array}$$

(72) $$\begin{array}{ccc} & = & g_{1}\left(\xi \right)+\sum_{j=1}^{m-1}g_{j}^{\left(1\right)}\left(\xi \right). \end{array}$$

 □

**Theorem** **4.** *We have the following relation:*

(73) $$\begin{array}{c} g_{m+1}^{\left(1\right)}\left(\xi \right)=\frac{1}{\xi }-\frac{m}{\xi }g_{m}^{\left(1\right)}\left(\xi \right) \end{array}$$

*for all m∈N and all ξ≥0.*

**Proof.**  See Appendix C.2. □

**Corollary** **4.** *For any m>1,*

(74) $$\begin{array}{c} E\left[\frac{1}{V}\right]=\frac{1-\lambda \;g_{m}^{\left(1\right)}\left(\lambda \right)}{m-1}. \end{array}$$

**Proof.**  This follows directly from (73) and (59). □

### 5.2. Properties of $h_{n}\left(\cdot \right)$

**Proposition** **10.** For any $n\in N^{\mathrm{odd}}$, the function $\xi \mapsto h_{n}\left(\xi \right)$ is monotonically strictly increasing and strictly concave ($\xi \in R_{0}^{+}$).

**Proof.**  From (A95) and (A98) we see that $h_{n}^{\left(1\right)}\left(\xi \right)>0$ and $h_{n}^{\left(2\right)}\left(\xi \right)<0$. □

**Proposition** **11.** For any ξ≥0, the function $n\mapsto h_{n}\left(\xi \right)$ is monotonically strictly increasing ($n\in N^{\mathrm{odd}}$).

**Proof.**  This follows directly from Theorem 1 and Proposition 2. □

**Proposition** **12.** For any $n\in N^{\mathrm{odd}}$, the function $\xi \mapsto h_{n}^{\left(1\right)}\left(\xi \right)$ is positive, monotonically strictly decreasing, and strictly convex ($\xi \in R_{0}^{+}$).

**Proof.**  The positivity and the monotonicity follow directly from (A95) and (A98). To see the convexity, we use (A99) to write

(75) $$\begin{array}{c} h_{n}^{\left(3\right)}\left(\xi \right)=\;e^{-\xi }\sum_{k=0}^{\infty }\frac{\xi^{k}}{k!}\;\frac{2}{\left(k+\frac{n}{2}\right)\left(k+1+\frac{n}{2}\right)\left(k+2+\frac{n}{2}\right)}, \end{array}$$

which is positive. □

**Proposition** **13.** For any ξ≥0, the function $n\mapsto h_{n}^{\left(1\right)}\left(\xi \right)$ is monotonically strictly decreasing ($n\in N^{\mathrm{odd}}$).

**Proof.**  This follows directly from Theorem 2 and Proposition 2. □

**Theorem** **5.** *For all $n\in N^{\mathrm{odd}}$, ξ≥0, and ℓ∈N, we have the following relations:*

(76a) $$\begin{array}{ccc} h_{n+2}\left(\xi \right) & = & h_{n}\left(\xi \right)+h_{n}^{\left(1\right)}\left(\xi \right), \end{array}$$

(76b) $$\begin{array}{ccc} h_{n+2}^{\left(\ell \right)}\left(\xi \right) & = & h_{n}^{\left(\ell \right)}\left(\xi \right)+h_{n}^{\left(\ell +1\right)}\left(\xi \right). \end{array}$$

**Proof.**  See Appendix C.1. □

**Corollary** **5.** *For any $n\in N^{\mathrm{odd}}$, n≥3,*

(77) $$\begin{array}{c} E\left[\frac{1}{U^{\left[n,\tau \right]}}\right]=\frac{1}{2}h_{n}\left(\frac{\tau }{2}\right)-\frac{1}{2}h_{n-2}\left(\frac{\tau }{2}\right). \end{array}$$

**Proof.**  This follows directly from (76a) and (58). □

**Corollary** **6.** *The $\ell^{th}$ derivative $h_{n}^{\left(\ell \right)}\left(\cdot \right)$ can be written with the help of either the first derivative $h_{n}^{\left(1\right)}\left(\cdot \right)$ or of $h_{n}\left(\cdot \right)$ in the following ways:*

(78) hn(ℓ)(ξ)=∑j=0ℓ−1(−1)j+ℓ−1ℓ−1jhn+2j(1)(ξ)

(79) =∑j=0ℓ(−1)j+ℓℓjhn+2j(ξ).

**Proof.**  We rewrite (76) as

(80) $$\begin{array}{c} h_{n}^{\left(\ell \right)}\left(\xi \right)=h_{n+2}^{\left(\ell -1\right)}\left(\xi \right)-h_{n}^{\left(\ell -1\right)}\left(\xi \right) \end{array}$$

(where $h_{n}^{\left(0\right)}$ is understood as being equivalent to $h_{n}$) and recursively apply this relation. □

**Corollary** **7.** *For all $n\in N^{\mathrm{odd}}$ and ξ≥0,*

(81) $$\begin{array}{ccc} h_{n}\left(\xi \right) & = & h_{1}\left(\xi \right)+\sum_{j=1}^{\frac{n-1}{2}}h_{2j-1}^{\left(1\right)}\left(\xi \right) \end{array}$$

(82) $$\begin{array}{ccc} & = & -\gamma -2\ln\left(2\right)+2\xi \cdot {}_{2}F_{2}\left(1,1;\frac{3}{2},2;-\xi \right)+\sum_{j=1}^{\frac{n-1}{2}}h_{2j-1}^{\left(1\right)}\left(\xi \right). \end{array}$$

**Proof.**  This follows by recursive application of (76a) in the same way as Corollary 3 follows from (63a). □

**Theorem** **6.** *We have the following relation:*

(83) $$\begin{array}{c} h_{n+2}^{\left(1\right)}\left(\xi \right)=\frac{1}{\xi }-\frac{n}{2\xi }h_{n}^{\left(1\right)}\left(\xi \right) \end{array}$$

*for all $n\in N^{\mathrm{odd}}$ and all ξ≥0.*

**Proof.**  See Appendix C.2. □

**Corollary** **8.** *For any $n\in N^{\mathrm{odd}}$, n≥3,*

(84) $$\begin{array}{c} E\left[\frac{1}{U}\right]=\frac{1-\frac{\tau }{2}\;h_{n}^{\left(1\right)}\left(\frac{\tau }{2}\right)}{n-2}. \end{array}$$

**Proof.**  This follows directly from (83) and (58). □

### 5.3. Additional Properties

**Proposition** **14.** *For all ξ≥0, if $n\in N^{\mathrm{odd}}$,*

(85) $$\begin{array}{c} h_{n}\left(\xi \right)\leq g_{\frac{n+1}{2}}\left(\xi \right), \end{array}$$

*and if $n\in N^{\mathrm{even}}$,*

(86) $$\begin{array}{c} g_{\frac{n}{2}}\left(\xi \right)\leq h_{n+1}\left(\xi \right). \end{array}$$

*Similarly, for all ξ≥0, if $n\in N^{\mathrm{odd}}$,*

(87) $$\begin{array}{c} h_{n}^{\left(1\right)}\left(\xi \right)\geq g_{\frac{n+1}{2}}^{\left(1\right)}\left(\xi \right), \end{array}$$

*and if $n\in N^{\mathrm{even}}$,*

(88) $$\begin{array}{c} g_{\frac{n}{2}}^{\left(1\right)}\left(\xi \right)\geq h_{n+1}^{\left(1\right)}\left(\xi \right). \end{array}$$

**Proof.**  The relations (85) and (86) follow from (54) and Proposition 2; and (87) and (88) follow from (58) and Proposition 2. See also Figure 1 and Figure 2 for a graphical representation of this relationship. □

**Lemma** **2.** For any m∈N, the function $\xi \mapsto g_{m}\left(\frac{1}{\xi }\right)$ is monotonically strictly decreasing and convex. Similarly, for any $n\in N^{\mathrm{odd}}$, the function $\xi \mapsto h_{n}\left(\frac{1}{\xi }\right)$ is monotonically strictly decreasing and convex.

**Proof.**  Since

(89) $$\begin{array}{c} \frac{\partial }{\partial \xi }g_{m}\left(\frac{1}{\xi }\right)=-\frac{1}{\xi^{2}}\;g_{m}^{\left(1\right)}\left(\frac{1}{\xi }\right) \end{array}$$

and because (by Proposition 8) $g_{m}^{\left(1\right)}\left(\cdot \right)>0$, we conclude that $g_{m}\left(\frac{1}{\xi }\right)$ is monotonically strictly decreasing.

To check convexity, we use Theorem 3 to rewrite (89) as

(90) $$\begin{array}{ccc} \frac{\partial }{\partial \xi }g_{m}\left(\frac{1}{\xi }\right) & = & -\frac{1}{\xi^{2}}\;g_{m+1}\left(\frac{1}{\xi }\right)+\frac{1}{\xi^{2}}\;g_{m}\left(\frac{1}{\xi }\right) \end{array}$$

such that

(91) $$\begin{array}{ccc} \frac{\partial^{2}}{\partial \xi^{2}}g_{m}\left(\frac{1}{\xi }\right) & = & \frac{2}{\xi^{3}}\;g_{m+1}\left(\frac{1}{\xi }\right)+\frac{1}{\xi^{4}}\;g_{m+1}^{\left(1\right)}\left(\frac{1}{\xi }\right)-\frac{2}{\xi^{3}}\;g_{m}\left(\frac{1}{\xi }\right)-\frac{1}{\xi^{4}}\;g_{m}^{\left(1\right)}\left(\frac{1}{\xi }\right) \end{array}$$

(92) $$\begin{array}{ccc} & = & \frac{2}{\xi^{3}}\;g_{m}^{\left(1\right)}\left(\frac{1}{\xi }\right)+\frac{1}{\xi^{4}}\left(\xi -m\xi \;g_{m}^{\left(1\right)}\left(\frac{1}{\xi }\right)\right)-\frac{1}{\xi^{4}}\;g_{m}^{\left(1\right)}\left(\frac{1}{\xi }\right) \end{array}$$

(93) $$\begin{array}{ccc} & = & g_{m}^{\left(1\right)}\left(\frac{1}{\xi }\right)\cdot \frac{2\xi -m\xi -1}{\xi^{4}}+\frac{1}{\xi^{3}} \end{array}$$

(94) $$\begin{array}{ccc} & \geq & \frac{1}{\frac{1}{\xi }+m}\cdot \frac{2\xi -m\xi -1}{\xi^{4}}+\frac{1}{\xi^{3}} \end{array}$$

(95) $$\begin{array}{ccc} & = & \frac{2}{\xi^{2}\left(m\xi +1\right)}>0. \end{array}$$

Here, in the second equality we use Theorems 3 and 4; and the inequality follows from the lower bound (105) in Theorem 8 below. (Note that while the derivation of the bounds in Section 6 do strongly rely on the properties derived in Section 5, the results of this Lemma 2 are not needed there.)

The derivation for $h_{n}\left(\frac{1}{\xi }\right)$ is completely analogous. In particular, using Theorems 5 and 6 one shows that

(96) $$\begin{array}{ccc} \frac{\partial^{2}}{\partial \xi^{2}}h_{n}\left(\frac{1}{\xi }\right) & = & h_{n}^{\left(1\right)}\left(\frac{1}{\xi }\right)\cdot \frac{2\xi -\frac{n}{2}\xi -1}{\xi^{4}}+\frac{1}{\xi^{3}} \end{array}$$

(97) $$\begin{array}{ccc} & \geq & \frac{1}{\frac{1}{\xi }+\frac{n}{2}}\cdot \frac{2\xi -\frac{n}{2}\xi -1}{\xi^{4}}+\frac{1}{\xi^{3}} \end{array}$$

(98) $$\begin{array}{ccc} & = & \frac{2}{\xi^{2}\left(\frac{n}{2}\xi +1\right)}>0, \end{array}$$

where the inequality follows from (117) in Theorem 10 below. □

## 6. Bounds

Finally, we derive some elementary upper and lower bounds on $g_{m}\left(\cdot \right)$ and $h_{n}\left(\cdot \right)$ and their first derivative.

### 6.1. Bounds on $g_{m}\left(\cdot \right)$ and $g_{m}^{\left(1\right)}\left(\cdot \right)$

**Theorem** **7.** *For any m∈N and $\xi \in R_{0}^{+}$, $g_{m}\left(\xi \right)$ is lower-bounded as follows:*

(99) $$\begin{array}{ccc} g_{m}\left(\xi \right) & \geq & \ln(\xi +m-1), \end{array}$$

(100) $$\begin{array}{ccc} g_{m}\left(\xi \right) & \geq & \ln(\xi +m)-\ln(m)+\psi (m), \end{array}$$

*and upper-bounded as follows:*

(101) $$\begin{array}{ccc} g_{m}\left(\xi \right) & \leq & \ln(\xi +m), \end{array}$$

(102) $$\begin{array}{ccc} g_{m}\left(\xi \right) & \leq & \frac{m+1}{m}\ln\left(\frac{\xi +m+1}{m+1}\right)+\psi \left(m\right). \end{array}$$

**Proof.**  See Appendix D.1. □

Note that the bounds (101) and (99) are tighter for larger values of ξ, and they are exact asymptotically when ξ→∞:

(103) $$\begin{array}{c} \lim_{\xi \to \infty }\left\{\ln(\xi +m)-\ln(\xi +m-1)\right\}=0. \end{array}$$

In contrast, the bounds (102) and (100) are better for small values of ξ and are exact for ξ=0:

(104) $$\begin{array}{c} \lim_{\xi ↓0}\frac{\frac{m+1}{m}\ln\left(\frac{\xi +m+1}{m+1}\right)+\psi \left(m\right)}{\ln(\xi +m)-\ln(m)+\psi (m)}=1. \end{array}$$

In general, the tightness of the bounds increases with increasing *m*.

**Figure 3:**
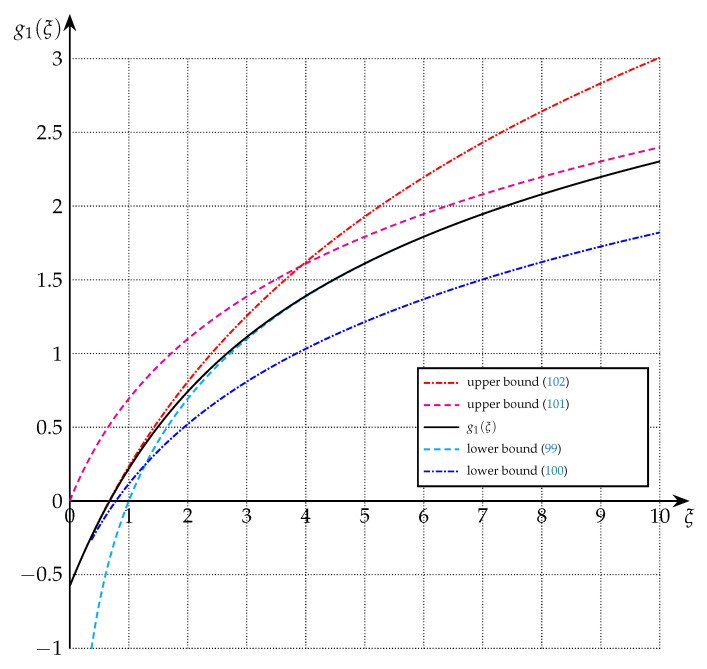
Upper and lower bounds on $g_{1}\left(\cdot \right)$ from Theorem 7 (m=1). For small ξ, (102) and (100) are tight, and while (101) and (99) are tight for larger ξ. In particular, (99) is extremely tight for ξ≥2, and (102) for ξ≤2.

**Figure 4:**
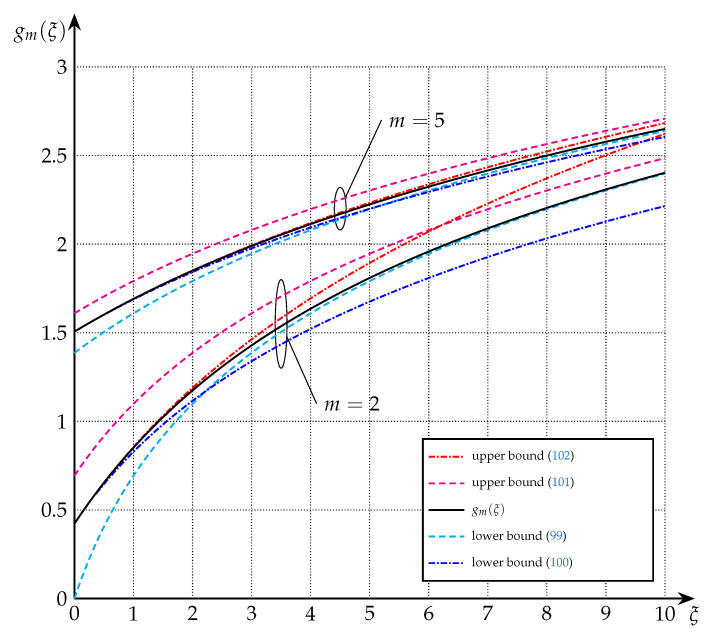
Upper and lower bounds on $g_{m}\left(\cdot \right)$ from Theorem 7 for m=2 and m=5.

The bounds of Theorem 7 are depicted in Figure 3 and Figure 4 for the cases of m=1, m=2, and m=5.

**Theorem** **8.** *For any m∈N and $\xi \in R_{0}^{+}$, $g_{m}^{\left(1\right)}\left(\xi \right)$ is lower-bounded as follows:*

(105) $$\begin{array}{c} g_{m}^{\left(1\right)}\left(\xi \right)\geq \frac{1}{\xi +m}, \end{array}$$

*and upper-bounded as follows:*

(106) $$\begin{array}{ccc} g_{m}^{\left(1\right)}\left(\xi \right) & \leq & \frac{m+1}{m(\xi +m+1)}, \end{array}$$

(107) $$\begin{array}{ccc} g_{m}^{\left(1\right)}\left(\xi \right) & \leq & \frac{1}{\xi +m-1}. \end{array}$$

**Proof.**  See Appendix D.1. □

Note that the lower bound (105) is exact for ξ=0 and asymptotically when ξ→∞. The upper bound (106) is tighter for small values of ξ and is exact for ξ=0, while (107) is better for larger values of ξ and is exact asymptotically when ξ→∞. Concretely, we have

(108) $$\begin{array}{c} \lim_{\xi \to \infty }\left\{\frac{1}{\xi +m-1}-\frac{1}{\xi +m}\right\}=0 \end{array}$$

and

(109) $$\begin{array}{c} \lim_{\xi ↓0}\frac{\frac{m+1}{m(\xi +m+1)}}{\frac{1}{\xi +m}}=1. \end{array}$$

In general, also here it holds that the tightness of the bounds increases with increasing *m*.

**Figure 5:**
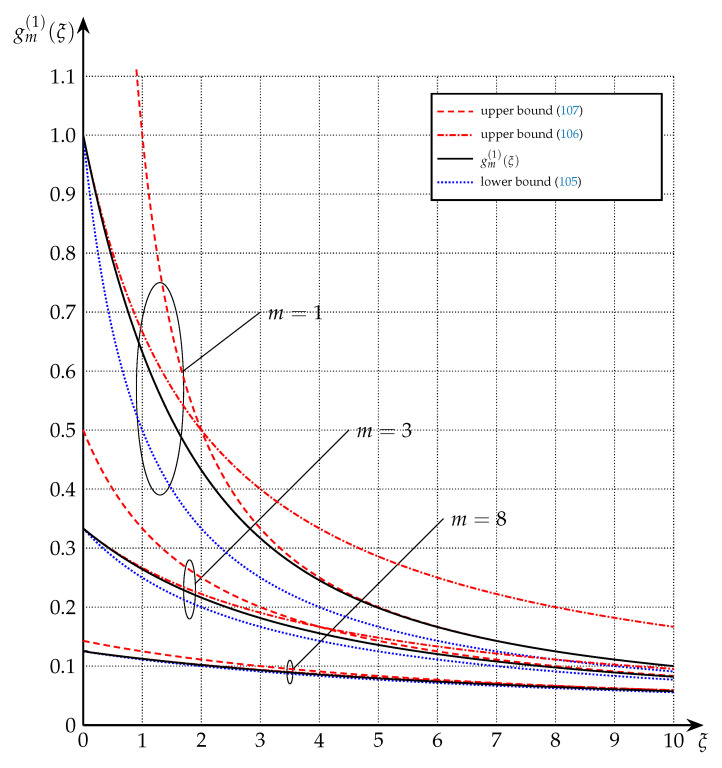
Upper and lower bounds on $g_{m}^{\left(1\right)}\left(\cdot \right)$ from Theorem 8 for m=1, m=3, and m=8. Note that for ξ<m+1 (106) is tighter than (107), while for ξ>m+1 (107) is tighter than (106).

The bounds of Theorem 8 are depicted in Figure 5 for the cases of m=1, m=3, and m=8.

### 6.2. Bounds on $h_{n}\left(\cdot \right)$ and $h_{n}^{\left(1\right)}\left(\cdot \right)$

**Theorem** **9.** *For any $\xi \in R_{0}^{+}$, $h_{n}\left(\xi \right)$ is lower-bounded as follows:*

(110a) $$\begin{array}{cccc} h_{n}\left(\xi \right) & \geq & \ln\left(\xi +\frac{n}{2}-1\right) & (n\in N^{\mathrm{odd}},\;n\geq 3), \end{array}$$

(110b) $$\begin{array}{cccc} h_{1}\left(\xi \right) & \geq & \ln\left(\xi +\frac{1}{2}\right)-\frac{2}{\xi }\left(1-\;e^{-\xi }\right) & (n=1). \end{array}$$

*Moreover, for any $n\in N^{\mathrm{odd}}$,*

(111) $$\begin{array}{ccc} h_{n}\left(\xi \right) & \geq & \ln\left(\xi +\frac{n}{2}\right)-\ln\left(\frac{n}{2}\right)+\psi \left(\frac{n}{2}\right). \end{array}$$

*For any $\xi \in R_{0}^{+}$ and any $n\in N^{\mathrm{odd}}$, $h_{n}\left(\xi \right)$ is upper-bounded as follows:*

(112) $$\begin{array}{ccc} h_{n}\left(\xi \right) & \leq & \ln\left(\xi +\frac{n}{2}\right), \end{array}$$

(113) $$\begin{array}{ccc} h_{n}\left(\xi \right) & \leq & \frac{n+2}{n}\ln\left(\frac{\xi +\frac{n}{2}+1}{\frac{n}{2}+1}\right)+\psi \left(\frac{n}{2}\right). \end{array}$$

**Proof.**  See Appendix D.2. □

Note that the bounds (112) and (110) are tighter for larger values of ξ, and they are exact asymptotically when ξ→∞:

(114) $$\begin{array}{c} \lim_{\xi \to \infty }\left\{\ln\left(\xi +\frac{n}{2}\right)-\ln\left(\xi +\frac{n}{2}-1\right)\right\}=0 \end{array}$$

and

(115) $$\begin{array}{c} \lim_{\xi \to \infty }\left\{\ln\left(\xi +\frac{1}{2}\right)-\ln\left(\xi +\frac{1}{2}\right)+\frac{2}{\xi }\left(1-\;e^{-\xi }\right)\right\}=0, \end{array}$$

respectively.

In contrast, the bounds (113) and (111) are better for small values of ξ and are exact for ξ=0:

(116) $$\begin{array}{c} \lim_{\xi ↓0}\frac{\frac{n+2}{n}\ln\left(\frac{\xi +\frac{n}{2}+1}{\frac{n}{2}+1}\right)+\psi \left(\frac{n}{2}\right)}{\ln\left(\xi +\frac{n}{2}\right)-\ln\left(\frac{n}{2}\right)+\psi \left(\frac{n}{2}\right)}=1. \end{array}$$

In general, the tightness of the bounds increases with increasing *n*.

**Figure 6:**
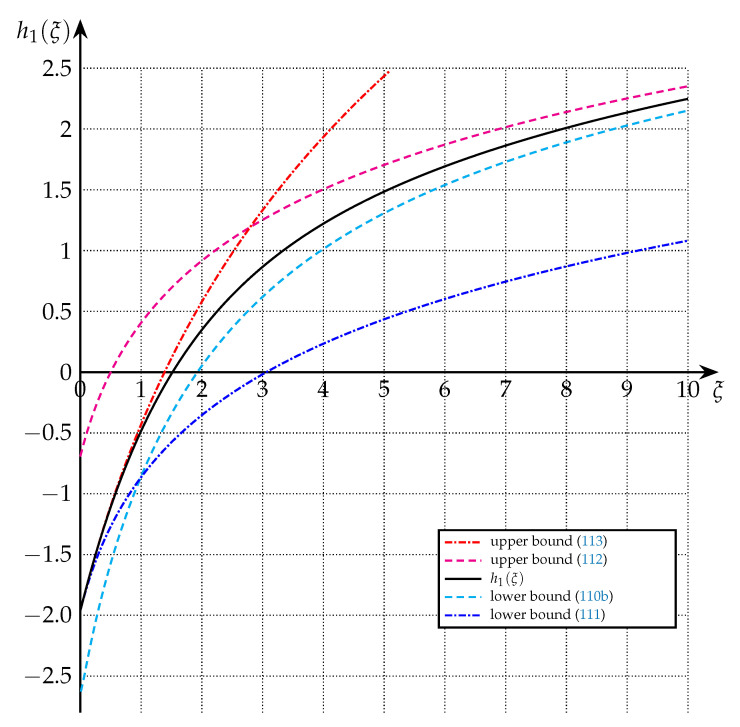
Upper and lower bounds on $h_{1}\left(\cdot \right)$ from Theorem 9 (n=1).

**Figure 7:**
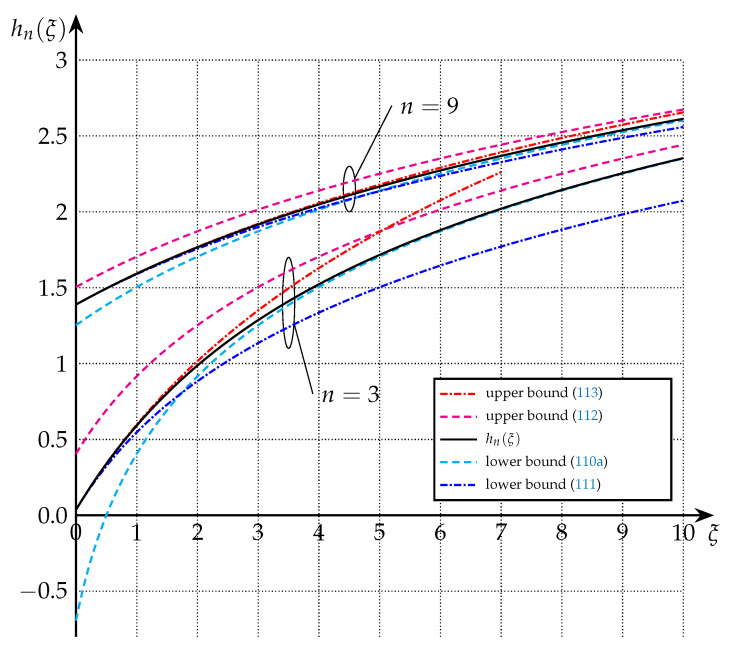
Upper and lower bounds on $h_{n}\left(\cdot \right)$ from Theorem 9 for n=3 and n=9.

The bounds of Theorem 9 are depicted in Figure 6 and Figure 7 for the cases of n=1, n=3, and n=9.

**Theorem** **10.** *For any $n\in N^{\mathrm{odd}}$ and $\xi \in R_{0}^{+}$, $h_{n}^{\left(1\right)}\left(\xi \right)$ is lower-bounded as follows:*

(117) $$\begin{array}{c} h_{n}^{\left(1\right)}\left(\xi \right)\geq \frac{1}{\xi +\frac{n}{2}}, \end{array}$$

*and upper-bounded as follows:*

(118) $$\begin{array}{ccc} h_{n}^{\left(1\right)}\left(\xi \right) & \leq & \frac{n+2}{n\left(\xi +\frac{n}{2}+1\right)}. \end{array}$$

*Moreover,*

(119a) $$\begin{array}{cccc} h_{n}^{\left(1\right)}\left(\xi \right) & \leq & \frac{1}{\xi +\frac{n}{2}-1} & (n\in N^{\mathrm{odd}},n\geq 3), \end{array}$$

(119b) $$\begin{array}{cccc} h_{1}^{\left(1\right)}\left(\xi \right) & \leq & \frac{2}{\xi }\left(1-\;e^{-\xi }\right)\leq \frac{2}{\xi } & (n=1). \end{array}$$

**Proof.**  See Appendix D.2. □

Note that the lower bound (117) is exact for ξ=0 and asymptotically when ξ→∞. The upper bound (118) is tighter for small values of ξ and is exact for ξ=0, while (119) is better for larger values of ξ and is exact asymptotically when ξ→∞. Concretely, we have

(120) $$\begin{array}{c} \lim_{\xi \to \infty }\left\{\frac{1}{\xi +\frac{n}{2}-1}-\frac{1}{\xi +\frac{n}{2}}\right\}=0 \end{array}$$

or

(121) $$\begin{array}{c} \lim_{\xi \to \infty }\left\{\frac{2}{\xi }-\frac{1}{\xi +\frac{n}{2}}\right\}=0, \end{array}$$

respectively, and

(122) $$\begin{array}{c} \lim_{\xi ↓0}\frac{\frac{n+2}{n\left(\xi +\frac{n}{2}+1\right)}}{\frac{1}{\xi +\frac{n}{2}}}=1. \end{array}$$

In the special case n=1, the improved version of (119b) is exact also for ξ=0, but it is still less tight for low ξ than (118).

In general, also here it holds that the tightness of the bounds increases with increasing *n*.

**Figure 8:**
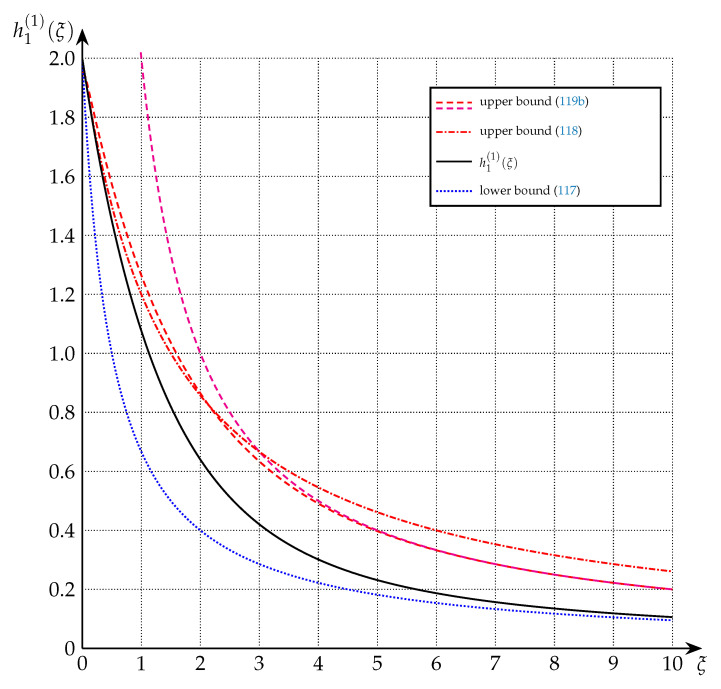
Upper and lower bounds on $h_{1}^{\left(1\right)}\left(\cdot \right)$ from Theorem 10 (n=1).

**Figure 9:**
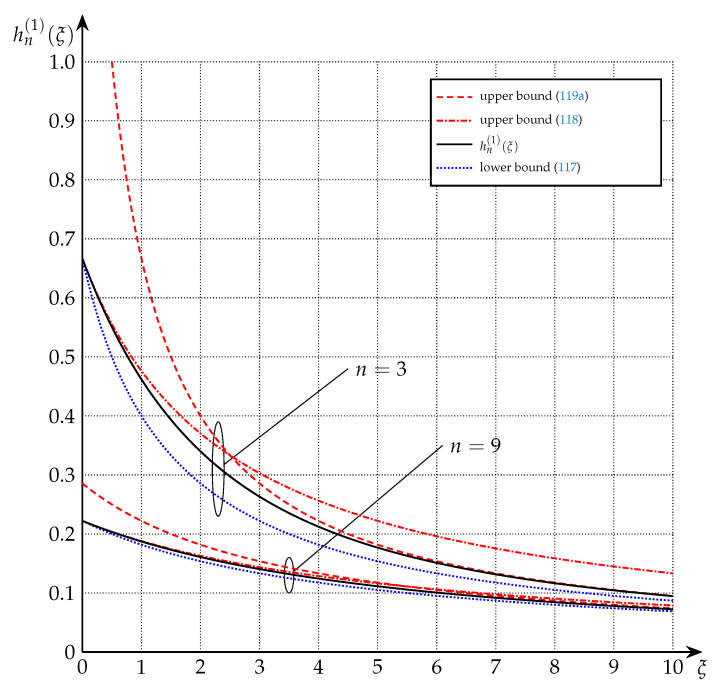
Upper and lower bounds on $h_{n}^{\left(1\right)}\left(\cdot \right)$ from Theorem 10 for n=3, and n=9. Note that for $\xi <\frac{n}{2}+1$ (118) is tighter than (119a), while for $\xi >\frac{n}{2}+1$ (119a) is tighter than (118).

These bounds are depicted in Figure 8 and Figure 9 for the cases n=1, n=3, and n=9.

## 7. Discussion

We have shown that the expected logarithm and the negative integer moments of a noncentral $\chi^{2}$-distributed RV can be expressed with the help of two families of functions $g_{m}\left(\cdot \right)$ and $h_{n}\left(\cdot \right)$, depending on whether the degrees of freedom are even or odd. While these two families of functions are very similar in many respects, they are actually surprisingly different in their description. The case of odd degrees of freedom thereby turns out to be quite a bit more complicated than the situation of even degrees of freedom (which explains why $g_{m}\left(\cdot \right)$ was defined in [1] already, while $h_{n}\left(\cdot \right)$ is newly introduced in this work).

We have also provided a whole new set of properties of both family of functions and derived new tight upper and lower bounds that are solely based on elementary functions.

It is intuitively pleasing that $U^{-\ell }$—being proportional to the ℓth derivative of ln(U)—has an expectation that is related to the $\ell^{th}$ derivative of the function describing the expectation of the logarithm.

The recently proposed trick of representing the logarithm by an integral [7] turned out to be very helpful in the proof of the continuity of the expected logarithm (see Appendix A.3). While in general very well behaved, the logarithmic function nevertheless is a fickle beast due to its unboundedness both at zero and infinity.

## Figures and Tables

**Figure A1:**
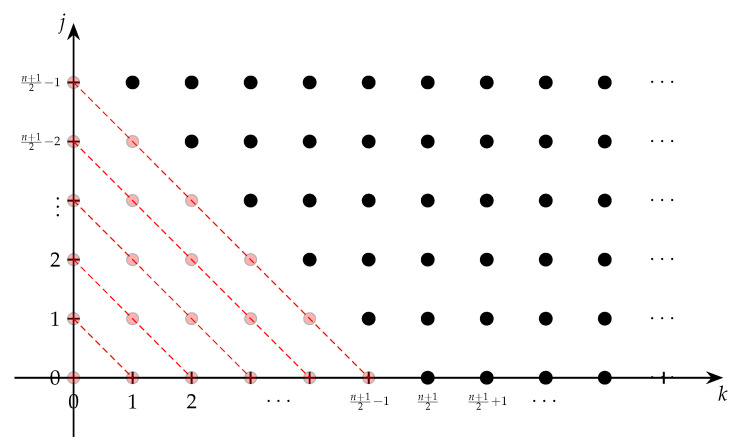
The black dots depict the values covered by the counters *k* and *j* in the double sum of S in (A67).

## Appendix A. Proofs of Section 2

### Appendix A.1. Proof of Proposition 1

We first look at the case τ=0. Using the PDF (8), we compute

(A1) $$\begin{array}{ccc} E\left[{\left(U^{\left[n,0\right]}\right)}^{-\ell }\right] & = & \int_{0}^{\infty }\frac{u^{-\ell +\frac{n}{2}-1}}{2^{\frac{n}{2}}\Gamma \left(\frac{n}{2}\right)}\;e^{-\frac{u}{2}}\;du \end{array}$$

(A2) $$\begin{array}{ccc} & = & \frac{\Gamma (\frac{n}{2}-\ell )}{2^{\ell }\;\Gamma \left(\frac{n}{2}\right)}<\infty , \end{array}$$

where (A2) follows from ([8], Eq. 3.381-4) as long as

(A3) $$\begin{array}{c} -\ell +\frac{n}{2}>0. \end{array}$$

On the other hand, if $\ell \geq \frac{n}{2}$, then (A1) can be bounded as follows:

(A4) $$\begin{array}{ccc} \int_{0}^{\infty }\frac{u^{-\ell +\frac{n}{2}-1}}{2^{\frac{n}{2}}\Gamma \left(\frac{n}{2}\right)}\;e^{-\frac{u}{2}}\;du & > & \frac{1}{2^{\frac{n}{2}}\Gamma \left(\frac{n}{2}\right)}\int_{0}^{1}u^{-\ell +\frac{n}{2}-1}\;e^{-\frac{u}{2}}\;du \end{array}$$

(A5) $$\begin{array}{ccc} & \geq & \frac{1}{2^{\frac{n}{2}}\Gamma \left(\frac{n}{2}\right)}\;e^{-\frac{1}{2}}\underset{=\infty }{\underbrace{\int_{0}^{1}u^{-\ell +\frac{n}{2}-1}\;du}}=\infty , \end{array}$$

where the first inequality holds because all terms in the integral are positive; where in the second inequality we have bounded

(A6) $$\begin{array}{c} \;e^{-\frac{u}{2}}\geq \;e^{-\frac{1}{2}},\;u\in \left[0,1\right]; \end{array}$$

and where the integral is infinite because from $\ell \geq \frac{n}{2}$ it follows that

(A7) $$\begin{array}{c} -\ell +\frac{n}{2}-1\leq -1. \end{array}$$

Next, assume that τ>0. Using the PDF (6) we write the negative moment as an integral and make a change of integration variable x≜τu:

(A8) $$\begin{array}{ccc} E\left[{\left(U^{\left[n,\tau \right]}\right)}^{-\ell }\right] & = & \int_{0}^{\infty }u^{-\ell }\cdot \frac{1}{2}{\left(\frac{u}{\tau }\right)}^{\frac{n-2}{4}}\;e^{-\frac{\tau +u}{2}}\;I_{n/2-1}\left(\sqrt{\tau u}\right)\;du \end{array}$$

(A9) $$\begin{array}{ccc} & = & \frac{1}{2}\tau^{\ell -\frac{n}{2}}\;e^{-\frac{\tau }{2}}\int_{0}^{\infty }x^{-\ell +\frac{n}{4}-\frac{1}{2}}\;e^{-\frac{x}{2\tau }}\;I_{n/2-1}\left(\sqrt{x}\right)\;dx \end{array}$$

(A10) $$\begin{array}{ccc} & = & \frac{1}{2}\tau^{\ell -\frac{n}{2}}\;e^{-\frac{\tau }{2}}\int_{0}^{\infty }x^{-\ell +\frac{n}{4}-\frac{1}{2}}\;e^{-\frac{x}{2\tau }}\sum_{k=0}^{\infty }\frac{x^{\frac{n}{4}-\frac{1}{2}+k}}{k!\;\Gamma \left(k+\frac{n}{2}\right)2^{\frac{n}{2}-1+2k}}\;dx \end{array}$$

(A11) $$\begin{array}{ccc} & = & 2^{-\frac{n}{2}}\tau^{\ell -\frac{n}{2}}\;e^{-\frac{\tau }{2}}\sum_{k=0}^{\infty }\frac{1}{k!\;\Gamma \left(k+\frac{n}{2}\right)2^{2k}}\int_{0}^{\infty }x^{k-\ell +\frac{n}{2}-1}\;e^{-\frac{x}{2\tau }}\;dx, \end{array}$$

where in (A10) we have relied on the series representation (7) of the modified Bessel function.

Now, if we again assume that $\ell <\frac{n}{2}$, we can use ([8], Eq. 3.381-4) to evaluate the integral and obtain

(A12) $$\begin{array}{ccc} E\left[{\left(U^{\left[n,\tau \right]}\right)}^{-\ell }\right] & = & 2^{-\frac{n}{2}}\tau^{\ell -\frac{n}{2}}\;e^{-\frac{\tau }{2}}\sum_{k=0}^{\infty }\frac{{\left(2\tau \right)}^{k-\ell +\frac{n}{2}}\;\Gamma \left(k+\frac{n}{2}-\ell \right)}{k!\;\Gamma \left(k+\frac{n}{2}\right)2^{2k}} \end{array}$$

(A13) $$\begin{array}{ccc} & = & 2^{-\ell }\;e^{-\frac{\tau }{2}}\sum_{k=0}^{\infty }\frac{\Gamma \left(k+\frac{n}{2}-\ell \right)}{k!\;\Gamma \left(k+\frac{n}{2}\right)}{\left(\frac{\tau }{2}\right)}^{k}. \end{array}$$

Using Γ(z+1)=zΓ(z) and noting that because $\frac{n}{2}-\ell >0$ we must have $\frac{n}{2}-\ell \geq \frac{1}{2}$, we bound

(A14) $$\begin{array}{ccc} \frac{\Gamma \left(k+\frac{n}{2}-\ell \right)}{\Gamma \left(k+\frac{n}{2}\right)} & = & \frac{\Gamma \left(k+\frac{n}{2}-\ell \right)}{\underset{\geq 1}{\underbrace{\left(k+\frac{n}{2}\right)}}\underset{\geq 1}{\underbrace{\left(k+\frac{n}{2}-1\right)}}\cdots \underset{\geq \frac{1}{2}}{\underbrace{\left(k+\frac{n}{2}-\ell \right)}}\Gamma \left(k+\frac{n}{2}-\ell \right)} \end{array}$$

(A15) $$\begin{array}{ccc} & \leq & 2. \end{array}$$

Thus, using the series expansion of the exponential function, we obtain from (A13),

(A16) $$\begin{array}{ccc} E\left[{\left(U^{\left[n,\tau \right]}\right)}^{-\ell }\right] & \leq & 2^{-\ell }\;e^{-\frac{\tau }{2}}\sum_{k=0}^{\infty }\frac{2}{k!}{\left(\frac{\tau }{2}\right)}^{k} \end{array}$$

(A17) $$\begin{array}{ccc} & = & 2^{-\ell +1}\;e^{-\frac{\tau }{2}}\;e^{\frac{\tau }{2}} \end{array}$$

(A18) $$\begin{array}{ccc} & = & 2^{-\ell +1}<\infty . \end{array}$$

On the other hand, if $\ell \geq \frac{n}{2}$, we bound (A10) by reducing the integral boundaries and by dropping all terms in the sum apart from k=0:

(A19) $$\begin{array}{ccc} E\left[{\left(U^{\left[n,\tau \right]}\right)}^{-\ell }\right] & > & 2^{-\frac{n}{2}}\tau^{\ell -\frac{n}{2}}\;e^{-\frac{\tau }{2}}\frac{1}{\Gamma \left(\frac{n}{2}\right)}\int_{0}^{1}x^{-\ell +\frac{n}{2}-1}\;e^{-\frac{x}{2\tau }}\;dx \end{array}$$

(A20) $$\begin{array}{ccc} & \geq & 2^{-\frac{n}{2}}\tau^{\ell -\frac{n}{2}}\;e^{-\frac{\tau }{2}-\frac{1}{2\tau }}\frac{1}{\Gamma \left(\frac{n}{2}\right)}\underset{=\infty }{\underbrace{\int_{0}^{1}x^{-\ell +\frac{n}{2}-1}\;dx}}=\infty , \end{array}$$

where the second inequality follows because

(A21) $$\begin{array}{c} \;e^{-\frac{x}{2\tau }}\geq \;e^{-\frac{1}{2\tau }},\;x\in \left[0,1\right], \end{array}$$

and where the integral is infinite because (A7) holds. This concludes the proof of Proposition 1.

### Appendix A.2. Proof of Proposition 2

We fix some τ≥0, two arbitrary natural numbers $n_{1},n_{2}\in N$ such that $n_{1}<n_{2}$, and some ℓ∈N such that $\ell <\frac{n_{2}}{2}$. We choose $\mu_{1}=\sqrt{\tau }$, $\mu_{2}=\cdots =\mu_{n_{2}}=0$, and let ${\left\{X_{k}\right\}}_{k=1}^{n_{2}}$ be IID $∼N_{R}\left(0,1\right)$. Then

(A22) $$\begin{array}{ccc} E\left[{\left(U^{\left[n_{2},\tau \right]}\right)}^{-\ell }\right] & = & E\left[{\left(\sum_{k=1}^{n_{2}}{\left(X_{k}+\mu_{k}\right)}^{2}\right)}^{-\ell }\right] \end{array}$$

(A23) $$\begin{array}{ccc} & = & E\left[{\left(\sum_{k=1}^{n_{1}}{\left(X_{k}+\mu_{k}\right)}^{2}+\sum_{k=n_{1}+1}^{n_{2}}{\left(X_{k}+\mu_{k}\right)}^{2}\right)}^{-\ell }\right] \end{array}$$

(A24) $$\begin{array}{ccc} & < & E\left[{\left(\sum_{k=1}^{n_{1}}{\left(X_{k}+\mu_{k}\right)}^{2}\right)}^{-\ell }\right] \end{array}$$

(A25) $$\begin{array}{ccc} & = & E\left[{\left(U^{\left[n_{1},\tau \right]}\right)}^{-\ell }\right], \end{array}$$

where the first equality follows from (5); the subsequent equality from splitting the sum into two parts; the subsequent inequality from the monotonicity of $\xi \mapsto \xi^{-\ell }$ and from dropping some terms that with probability 1 are positive; and the final equality again from (5). This proves the (decreasing) monotonicity of the negative integer moment in *n*.

The derivation of the (increasing) monotonicity of the expected logarithm is identical apart from that we rely on the (increasing) monotonicity of the logarithm instead of the (decreasing) monotonicity of $\xi \mapsto \xi^{-\ell }$.

### Appendix A.3. Proof of Proposition 3

To prove that $E_{}\left[\ln\left(U^{\left[n,\tau \right]}\right)\right]$ is continuous in τ we need to show that we are allowed to swap the order of a limit on τ and the expectation. This could be done using the Monotone Convergence Theorem or the Dominated Convergence Theorem [13]. Unfortunately, neither can be applied directly because ξ↦ln(ξ) is not nonnegative and unbounded both above and below.

Instead we rely on a trick recently presented in [7] that allows us to write the expected logarithm with the help of the MGF:

(A26) $$\begin{array}{ccc} E\left[\ln(U)\right] & = & \int_{0}^{\infty }\left(\;e^{-t}-E\left[\;e^{-tU}\right]\right)\frac{1}{t}\;dt. \end{array}$$

So, using the MGF of $U^{\left[n,\tau \right]}$,

(A27) $$\begin{array}{c} E\left[\;e^{-tU^{\left[n,\tau \right]}}\right]=\frac{\;e^{-\frac{\tau t}{1+2t}}}{{\left(1+2t\right)}^{\frac{n}{2}}},\;t\geq 0, \end{array}$$

we have

(A28) $$\begin{array}{ccc} E\left[\ln\left(U^{\left[n,\tau \right]}\right)\right] & = & \int_{0}^{\infty }\left(\;e^{-t}-\frac{\;e^{-\frac{\tau t}{1+2t}}}{{\left(1+2t\right)}^{\frac{n}{2}}}\right)\frac{1}{t}\;dt. \end{array}$$

We use this to prove continuity as follows. Assume that 0≤τ≤K for some arbitrary large, but finite constant K. Then

(A29) $$\begin{array}{ccc} \lim_{\epsilon ↓0}E\left[\ln\left(U^{\left[n,\tau +\epsilon \right]}\right)\right] & = & \lim_{\epsilon ↓0}\int_{0}^{\infty }\left(\;e^{-t}-\frac{\;e^{-\frac{(\tau +\epsilon )t}{1+2t}}}{{\left(1+2t\right)}^{\frac{n}{2}}}\right)\frac{1}{t}\;dt \end{array}$$

(A30) $$\begin{array}{ccc} & = & \int_{0}^{\infty }\lim_{\epsilon ↓0}\left(\;e^{-t}-\frac{\;e^{-\frac{(\tau +\epsilon )t}{1+2t}}}{{\left(1+2t\right)}^{\frac{n}{2}}}\right)\frac{1}{t}\;dt \end{array}$$

(A31) $$\begin{array}{ccc} & = & \int_{0}^{\infty }\left(\;e^{-t}-\frac{\;e^{-\frac{\tau t}{1+2t}}}{{\left(1+2t\right)}^{\frac{n}{2}}}\right)\frac{1}{t}\;dt \end{array}$$

(A32) $$\begin{array}{ccc} & = & E\left[\ln\left(U^{\left[n,\tau \right]}\right)\right]. \end{array}$$

It only remains to justify the swap of integration and limit in (A30). To that goal we rely on the Dominated Convergence Theorem applied to the function

(A33) $$\begin{array}{c} f_{\tau ,n}\left(t\right)≜\frac{\;e^{-t}}{t}-\frac{\;e^{-\frac{\tau t}{1+2t}}}{t{\left(1+2t\right)}^{\frac{n}{2}}}. \end{array}$$

Note that if

(A34) $$\begin{array}{c} \frac{\;e^{-t}}{t}\geq \frac{\;e^{-\frac{\tau t}{1+2t}}}{t{\left(1+2t\right)}^{\frac{n}{2}}}, \end{array}$$

we have from τ≤K

(A35) $$\begin{array}{ccc} |f_{\tau ,n}\left(t\right)|=f_{\tau ,n}\left(t\right) & = & \frac{\;e^{-t}}{t}-\frac{\;e^{-\frac{\tau t}{1+2t}}}{t{\left(1+2t\right)}^{\frac{n}{2}}} \end{array}$$

(A36) $$\begin{array}{ccc} & \leq & \frac{\;e^{-t}}{t}-\frac{\;e^{-Kt}}{t{\left(1+2t\right)}^{\frac{n}{2}}}. \end{array}$$

On the other hand, if

(A37) $$\begin{array}{c} \frac{\;e^{-t}}{t}<\frac{\;e^{-\frac{\tau t}{1+2t}}}{t{\left(1+2t\right)}^{\frac{n}{2}}}, \end{array}$$

we have

(A38) $$\begin{array}{ccc} |f_{\tau ,n}\left(t\right)|=-f_{\tau ,n}\left(t\right) & = & \frac{\;e^{-\frac{\tau t}{1+2t}}}{t{\left(1+2t\right)}^{\frac{n}{2}}}-\frac{\;e^{-t}}{t} \end{array}$$

(A39) $$\begin{array}{ccc} & \leq & \frac{1}{t{\left(1+2t\right)}^{\frac{1}{2}}}-\frac{\;e^{-t}}{t}. \end{array}$$

Thus, for all t≥0,

(A40) $$\begin{array}{ccc} |f_{\tau ,n}\left(t\right)| & \leq & \max\left\{\frac{\;e^{-t}}{t}-\frac{\;e^{-Kt}}{t{\left(1+2t\right)}^{\frac{n}{2}}},\frac{1}{t\sqrt{1+2t}}-\frac{\;e^{-t}}{t}\right\}≜F_{n}\left(t\right). \end{array}$$

Since both functions in the maximum of $F_{n}\left(t\right)$ are nonnegative for all t≥0, we can bound the maximum by the sum:

(A41) $$\begin{array}{ccc} F_{n}\left(t\right) & \leq & \frac{\;e^{-t}}{t}-\frac{\;e^{-Kt}}{t{\left(1+2t\right)}^{\frac{n}{2}}}+\frac{1}{t\sqrt{1+2t}}-\frac{\;e^{-t}}{t} \end{array}$$

(A42) $$\begin{array}{ccc} & = & \frac{1}{t\sqrt{1+2t}}-\frac{\;e^{-Kt}}{t{\left(1+2t\right)}^{\frac{n}{2}}} \end{array}$$

(A43) $$\begin{array}{ccc} & = & \frac{{\left(1+2t\right)}^{\frac{n-1}{2}}-\;e^{-Kt}}{t{\left(1+2t\right)}^{\frac{n}{2}}} \end{array}$$

and therefore

(A44) $$\begin{array}{ccc} \int_{0}^{\infty }F_{n}\left(t\right)\;dt & \leq & \int_{0}^{\infty }\frac{{\left(1+2t\right)}^{\frac{n-1}{2}}-\;e^{-Kt}}{t{\left(1+2t\right)}^{\frac{n}{2}}}\;dt<\infty , \end{array}$$

where the finiteness of the integral is obvious once we realize that the integrand is finite for all t≥0, in particular,

(A45) $$\begin{array}{c} \lim_{t↓0}\frac{{\left(1+2t\right)}^{\frac{n-1}{2}}-\;e^{-Kt}}{t{\left(1+2t\right)}^{\frac{n}{2}}}=K+n-1, \end{array}$$

and that for t≫1, the integrand grows like $t^{-\frac{3}{2}}$. Thus, all conditions needed for the Dominated Convergence Theorem are satisfied and the swap in (A30) is proven.

## Appendix B. Derivations of the Main Results (Theorems 1 and 2)

### Appendix B.1. Odd Degrees of Freedom

#### Appendix B.1.1. Expected Logarithm

Let *n* be odd and assume first τ=0. Taking the PDF (8) and using ([8], Eq. 4.352-1), we obtain

(A46) $$\begin{array}{ccc} E\left[\ln(U)\right] & = & \int_{0}^{\infty }\ln\left(u\right)\cdot \frac{1}{2^{\frac{n}{2}}\;\Gamma \left(\frac{n}{2}\right)}\;u^{\frac{n}{2}-1}\;e^{-\frac{u}{2}}\;du=\psi \left(\frac{n}{2}\right)+\ln\left(2\right), \end{array}$$

which proves the result for τ=0.

For τ>0, we need to evaluate the following integral:

(A47) $$\begin{array}{ccc} E\left[\ln(U)\right] & = & \int_{0}^{\infty }\ln\left(u\right)\cdot \frac{1}{2}{\left(\frac{u}{\tau }\right)}^{\frac{n-2}{4}}\;e^{-\frac{\tau +u}{2}}I_{\frac{n}{2}-1}\left(\sqrt{\tau u}\right)\;du. \end{array}$$

Expressing $I_{\frac{n}{2}-1}\left(\cdot \right)$ as the power series (7) we obtain:

(A48) $$\begin{array}{ccc} E\left[\ln(U)\right] & = & \int_{0}^{\infty }\ln\left(u\right)\;\frac{1}{2}\tau^{\frac{1}{2}-\frac{n}{4}}\;e^{-\frac{\tau }{2}-\frac{u}{2}}\;u^{\frac{n}{4}-\frac{1}{2}}\sum_{k=0}^{\infty }\frac{\tau^{k+\frac{n}{4}-\frac{1}{2}}\;u^{k+\frac{n}{4}-\frac{1}{2}}}{k!\;\Gamma \left(k+\frac{n}{2}\right)\;2^{2k+\frac{n}{2}-1}}\;du \end{array}$$

(A49) $$\begin{array}{ccc} & = & \int_{0}^{\infty }\ln\left(u\right)\;\frac{1}{2}\;e^{-\frac{\tau }{2}}\sum_{k=0}^{\infty }\frac{{\left(\frac{\tau }{2}\right)}^{k}}{k!\;\Gamma \left(k+\frac{n}{2}\right)}\;{\left(\frac{u}{2}\right)}^{k+\frac{n}{2}-1}\;e^{-\frac{u}{2}}\;du \end{array}$$

(A50) $$\begin{array}{ccc} & = & \;e^{-\frac{\tau }{2}}\sum_{k=0}^{\infty }\frac{{\left(\frac{\tau }{2}\right)}^{k}}{k!\;\Gamma \left(k+\frac{n}{2}\right)\;2^{k+\frac{n}{2}}}\int_{0}^{\infty }\ln\left(u\right)\;u^{k+\frac{n}{2}-1}\;e^{-\frac{u}{2}}\;du \end{array}$$

(A51) $$\begin{array}{ccc} & = & \;e^{-\frac{\tau }{2}}\sum_{k=0}^{\infty }\frac{1}{k!}{\left(\frac{\tau }{2}\right)}^{k}\left[\psi \left(k+\frac{n}{2}\right)+\ln\left(2\right)\right] \end{array}$$

(A52) $$\begin{array}{ccc} & = & \;e^{-\frac{\tau }{2}}\sum_{k=0}^{\infty }\frac{1}{k!}{\left(\frac{\tau }{2}\right)}^{k}\left[-\gamma -\ln\left(2\right)+\sum_{j=1}^{\frac{n-1}{2}+k}\frac{1}{j-\frac{1}{2}}\right] \end{array}$$

(A53) $$\begin{array}{ccc} & = & -\gamma -\ln\left(2\right)+\;e^{-\frac{\tau }{2}}\sum_{k=0}^{\infty }\frac{1}{k!}{\left(\frac{\tau }{2}\right)}^{k}\sum_{j=1}^{\frac{n-1}{2}+k}\frac{1}{j-\frac{1}{2}}. \end{array}$$

Here the interchange of summation and integral in (A50) is valid because we show in Appendix E that the sum converges uniformly for all u≥0; in (A51) we use the result from (A46) with $\frac{n}{2}$ replaced by $k+\frac{n}{2}$; (A52) follows from (51); and the last equality (A53) from the series expansion of the exponential function. We introduce the shorthand

(A54) $$\begin{array}{c} \xi ≜\frac{\tau }{2} \end{array}$$

and define the function

(A55) $$\begin{array}{c} {\tilde{h}}_{n}\left(\xi \right)≜-\gamma -2\ln\left(2\right)+\;e^{-\xi }\sum_{k=0}^{\infty }\frac{\xi^{k}}{k!}\sum_{j=1}^{\frac{n-1}{2}+k}\frac{1}{j-\frac{1}{2}}, \end{array}$$

such that

(A56) $$\begin{array}{c} E\left[\ln(U)\right]=\ln\left(2\right)+{\tilde{h}}_{n}\left(\frac{\tau }{2}\right). \end{array}$$

The proof will be concluded once we show that in fact ${\tilde{h}}_{n}\left(\xi \right)=h_{n}\left(\xi \right)$.

To that goal, we compute the derivative of (A55) by interchanging the derivative and the infinite summation (which again is valid due to uniform convergence proven in Appendix E):

(A57) $$\begin{array}{ccc} {\tilde{h}}_{n}^{\left(1\right)}\left(\xi \right) & = & \sum_{k=0}^{\infty }\frac{1}{k!}\sum_{j=1}^{\frac{n-1}{2}+k}\frac{1}{j-\frac{1}{2}}\left(-\;e^{-\xi }\xi^{k}+\;e^{-\xi }k\xi^{k-1}\right) \end{array}$$

(A58) $$\begin{array}{ccc} & = & \;e^{-\xi }\sum_{k=1}^{\infty }\sum_{j=1}^{\frac{n-1}{2}+k}\frac{\xi^{k-1}}{\left(k-1\right)!\;\left(j-\frac{1}{2}\right)}-\;e^{-\xi }\sum_{k=0}^{\infty }\sum_{j=1}^{\frac{n-1}{2}+k}\frac{\xi^{k}}{k!\;\left(j-\frac{1}{2}\right)} \end{array}$$

(A59) $$\begin{array}{ccc} & = & \;e^{-\xi }\sum_{k=0}^{\infty }\sum_{j=1}^{\frac{n-1}{2}+k+1}\frac{\xi^{k}}{k!\;\left(j-\frac{1}{2}\right)}-\;e^{-\xi }\sum_{k=0}^{\infty }\sum_{j=1}^{\frac{n-1}{2}+k}\frac{\xi^{k}}{k!\;\left(j-\frac{1}{2}\right)} \end{array}$$

(A60) $$\begin{array}{ccc} & = & \;e^{-\xi }\sum_{k=0}^{\infty }\frac{\xi^{k}}{k!\;\left(\frac{n-1}{2}+k+1-\frac{1}{2}\right)} \end{array}$$

(A61) $$\begin{array}{ccc} & = & \;e^{-\xi }\sum_{k=0}^{\infty }\frac{1}{k!\;\left(\frac{n}{2}+k\right)}\;\xi^{k} \end{array}$$

(A62) $$\begin{array}{ccc} & = & \frac{\;e^{-\xi }}{\xi^{\frac{n+1}{2}}}\sum_{\ell =\frac{n+1}{2}}^{\infty }\frac{1}{\left(\ell -\frac{n+1}{2}\right)!\;\left(\ell -\frac{1}{2}\right)}\;\xi^{\ell }. \end{array}$$

Here, in (A59) we shift *k* by −1 in the first sum; and (A62) follows from the substitution $\ell ≜k+\frac{n+1}{2}$.

Using the relation

(A63) $$\begin{array}{ccc} \sum_{j=0}^{\frac{n+1}{2}-1}\frac{{\left(-1\right)}^{j}}{\Gamma \left(j+\frac{1}{2}\right)\;\left(\ell -j\right)!} & = & \frac{-{\left(-1\right)}^{\frac{n+1}{2}}}{\left(\ell -\frac{1}{2}\right)\;\Gamma \left(\frac{n}{2}\right)\;\left(\ell -\frac{n+1}{2}\right)!}-\frac{1}{2\sqrt{\pi }\left(\ell -\frac{1}{2}\right)\;\ell !}, \end{array}$$

we thus obtain from (A62)

(A64) $$\begin{array}{ccc} {\tilde{h}}_{n}^{\left(1\right)}\left(\xi \right) & = & -\frac{\;e^{-\xi }\;{\left(-1\right)}^{\frac{n+1}{2}}\;\Gamma \left(\frac{n}{2}\right)}{\xi^{\frac{n+1}{2}}}\sum_{\ell =\frac{n+1}{2}}^{\infty }\frac{-{\left(-1\right)}^{\frac{n+1}{2}}}{\Gamma \left(\frac{n}{2}\right)\;\left(\ell -\frac{n+1}{2}\right)!\;\left(\ell -\frac{1}{2}\right)}\;\xi^{\ell } \end{array}$$

(A65) $$\begin{array}{ccc} & = & -\frac{\;e^{-\xi }\;{\left(-1\right)}^{\frac{n+1}{2}}\;\Gamma \left(\frac{n}{2}\right)}{\xi^{\frac{n+1}{2}}}\sum_{\ell =\frac{n+1}{2}}^{\infty }\left(\sum_{j=0}^{\frac{n+1}{2}-1}\frac{{\left(-1\right)}^{j}}{\Gamma \left(j+\frac{1}{2}\right)\;\left(\ell -j\right)!}+\frac{1}{2\sqrt{\pi }\left(\ell -\frac{1}{2}\right)\;\ell !}\right)\xi^{\ell } \end{array}$$

(A66) $$\begin{array}{ccc} & = & -\frac{\;e^{-\xi }\;{\left(-1\right)}^{\frac{n+1}{2}}\;\Gamma \left(\frac{n}{2}\right)}{\xi^{\frac{n+1}{2}}}(\underset{S}{\underbrace{\sum_{\ell =\frac{n+1}{2}}^{\infty }\sum_{j=0}^{\frac{n+1}{2}-1}\frac{{\left(-1\right)}^{j}}{\Gamma \left(j+\frac{1}{2}\right)\;\left(\ell -j\right)!}\;\xi^{\ell }}}+\frac{1}{2\sqrt{\pi }}\sum_{\ell =\frac{n+1}{2}}^{\infty }\frac{1}{\left(\ell -\frac{1}{2}\right)\;\ell !}\;\xi^{\ell }). \end{array}$$

We next swap the order of the sums in the term S and shift the counter *ℓ* by *j*, i.e., k≜ℓ−j:

(A67) $$\begin{array}{ccc} S & = & \sum_{j=0}^{\frac{n+1}{2}-1}\sum_{k=\frac{n+1}{2}-j}^{\infty }\frac{{\left(-1\right)}^{j}}{\Gamma \left(j+\frac{1}{2}\right)\;k!}\;\xi^{k+j}. \end{array}$$

Now, the counters *k* and *j* cover the values shown by the black dots in Figure A1.

We investigate the missing “triangle” of red dots, where we reorder the double sum to have an inner sum going along the “diagonals” (see again Figure A1) and an outer sum counting the diagonals:

(A68) $$\begin{array}{ccc} \sum_{k=0}^{\frac{n+1}{2}-1}\sum_{j=0}^{\frac{n+1}{2}-1-k}\frac{{\left(-1\right)}^{j}}{\Gamma \left(j+\frac{1}{2}\right)\;k!}\;\xi^{k+j} & = & \sum_{\ell =0}^{\frac{n+1}{2}-1}\sum_{t=0}^{\ell }\frac{{\left(-1\right)}^{\ell -t}}{\Gamma \left(\ell -t+\frac{1}{2}\right)\;t!}\;\xi^{\ell } \end{array}$$

(A69) $$\begin{array}{ccc} & = & \sum_{\ell =0}^{\frac{n+1}{2}-1}{\left(-1\right)}^{\ell }\;\xi^{\ell }\sum_{t=0}^{\ell }\frac{{\left(-1\right)}^{t}}{\Gamma \left(\ell -t+\frac{1}{2}\right)\;t!} \end{array}$$

(A70) $$\begin{array}{ccc} & = & \sum_{\ell =0}^{\frac{n+1}{2}-1}{\left(-1\right)}^{\ell }\;\xi^{\ell }\;\frac{{\left(-1\right)}^{\ell +1}}{2\sqrt{\pi }\left(\ell -\frac{1}{2}\right)\;\ell !} \end{array}$$

(A71) $$\begin{array}{ccc} & = & -\frac{1}{2\sqrt{\pi }}\sum_{\ell =0}^{\frac{n+1}{2}-1}\frac{1}{\left(\ell -\frac{1}{2}\right)\;\ell !}\;\xi^{\ell }, \end{array}$$

where in (A68) we set ℓ=k+j (in a diagonal the sum of *k* and *j* is constant!) and t=k; and (A70) follows from

(A72) $$\begin{array}{c} \sum_{t=0}^{\ell }\frac{{\left(-1\right)}^{t}}{\Gamma \left(\ell -t+\frac{1}{2}\right)\;t!}=\frac{{\left(-1\right)}^{\ell +1}}{2\sqrt{\pi }\left(\ell -\frac{1}{2}\right)\;\ell !}. \end{array}$$

Thus, we can rewrite S in (A67) as follows:

(A73) $$\begin{array}{ccc} S & = & \sum_{j=0}^{\frac{n+1}{2}-1}\sum_{k=0}^{\infty }\frac{{\left(-1\right)}^{j}}{\Gamma \left(j+\frac{1}{2}\right)\;k!}\;\xi^{k+j}+\frac{1}{2\sqrt{\pi }}\sum_{\ell =0}^{\frac{n+1}{2}-1}\frac{1}{\left(\ell -\frac{1}{2}\right)\;\ell !}\;\xi^{\ell }, \end{array}$$

and we therefore obtain from (A66)

(A74) $$\begin{array}{ccc} {\tilde{h}}_{n}^{\left(1\right)}\left(\xi \right) & = & -\frac{\;e^{-\xi }\;{\left(-1\right)}^{\frac{n+1}{2}}\;\Gamma \left(\frac{n}{2}\right)}{\xi^{\frac{n+1}{2}}}\left(\sum_{j=0}^{\frac{n+1}{2}-1}\sum_{k=0}^{\infty }\frac{{\left(-1\right)}^{j}}{\Gamma \left(j+\frac{1}{2}\right)\;k!}\;\xi^{k+j}+\frac{1}{2\sqrt{\pi }}\sum_{\ell =0}^{\infty }\frac{1}{\left(\ell -\frac{1}{2}\right)\;\ell !}\;\xi^{\ell }\right) \end{array}$$

(A75) $$\begin{array}{ccc} & = & \frac{\;e^{-\xi }\;{\left(-1\right)}^{\frac{n+1}{2}-1}\;\Gamma \left(\frac{n}{2}\right)}{\xi^{\frac{n+1}{2}}}(\sum_{j=0}^{\frac{n+1}{2}-1}\frac{{\left(-1\right)}^{j}\;\xi^{j}}{\Gamma \left(j+\frac{1}{2}\right)}\underset{=\;\;e^{\xi }}{\underbrace{\sum_{k=0}^{\infty }\frac{\xi^{k}}{k!}}}+\sqrt{\xi }\mathrm{erfi}\left(\sqrt{\xi }\right)-\frac{1}{\sqrt{\pi }}\;e^{\xi }) \end{array}$$

(A76) $$\begin{array}{ccc} & = & \frac{{\left(-1\right)}^{\frac{n+1}{2}-1}\;\Gamma \left(\frac{n}{2}\right)}{\xi^{\frac{n}{2}}}\;e^{-\xi }\mathrm{erfi}\left(\sqrt{\xi }\right)+\frac{{\left(-1\right)}^{\frac{n+1}{2}-1}\;\Gamma \left(\frac{n}{2}\right)}{\xi^{\frac{n+1}{2}}}\sum_{j=1}^{\frac{n+1}{2}-1}\frac{{\left(-1\right)}^{j}\;\xi^{j}}{\Gamma \left(j+\frac{1}{2}\right)}. \end{array}$$

Here, in the last equality we used that $\Gamma \left(\frac{1}{2}\right)=\sqrt{\pi }$, and (A75) can be shown as follows:

(A77) $$\begin{array}{ccc} \frac{1}{2\sqrt{\pi }}\sum_{\ell =0}^{\infty }\frac{1}{\left(\ell -\frac{1}{2}\right)\;\ell !}\;\xi^{\ell } & = & \frac{1}{\sqrt{\pi }}\sum_{\ell =0}^{\infty }\frac{\ell -\ell +\frac{1}{2}}{\left(\ell -\frac{1}{2}\right)\;\ell !}\;\xi^{\ell } \end{array}$$

(A78) $$\begin{array}{ccc} & = & \frac{1}{\sqrt{\pi }}\sum_{\ell =0}^{\infty }\left(\frac{\ell }{\left(\ell -\frac{1}{2}\right)\;\ell !}-\frac{\ell -\frac{1}{2}}{\left(\ell -\frac{1}{2}\right)\;\ell !}\right)\xi^{\ell } \end{array}$$

(A79) $$\begin{array}{ccc} & = & \frac{1}{\sqrt{\pi }}\sum_{\ell =1}^{\infty }\frac{1}{\left(\ell -\frac{1}{2}\right)\;\left(\ell -1\right)!}\;\xi^{\ell }-\frac{1}{\sqrt{\pi }}\sum_{\ell =0}^{\infty }\frac{1}{\ell !}\;\xi^{\ell } \end{array}$$

(A80) $$\begin{array}{ccc} & = & \frac{\xi^{\frac{1}{2}}}{\sqrt{\pi }}\sum_{\ell =0}^{\infty }\frac{1}{\left(\ell +\frac{1}{2}\right)\;\ell !}\;\xi^{\ell +\frac{1}{2}}-\frac{1}{\sqrt{\pi }}\;e^{\xi } \end{array}$$

(A81) $$\begin{array}{ccc} & = & \sqrt{\xi }\mathrm{erfi}\left(\sqrt{\xi }\right)-\frac{1}{\sqrt{\pi }}\;e^{\xi }, \end{array}$$

where (A80) follows from the series expansion of the exponential function and (A81) from the series expansion of the imaginary error function [14].

It thus remains to integrate the expression (A76). We only attempt this for the case n=1. Using the substitution $z=\sqrt{\xi }$, we obtain:

(A82) $$\begin{array}{ccc} {\tilde{h}}_{1}\left(\xi \right) & = & \int \sqrt{\frac{\pi }{\xi }}\;e^{-\xi }\mathrm{erfi}\left(\sqrt{\xi }\right)\;d\xi \end{array}$$

(A83) $$\begin{array}{ccc} & = & 4\int \frac{\sqrt{\pi }}{2}\;e^{-z^{2}}\mathrm{erfi}\left(z\right)\;dz \end{array}$$

(A84) $$\begin{array}{ccc} & = & 4\int D(z)\;dz \end{array}$$

(A85) $$\begin{array}{ccc} & = & 2z^{2}\cdot {}_{2}F_{2}\left(1,1;\frac{3}{2},2;-z^{2}\right)+c \end{array}$$

(A86) $$\begin{array}{ccc} & = & 2\xi \cdot {}_{2}F_{2}\left(1,1;\frac{3}{2},2;-\xi \right)+c \end{array}$$

where we used the indefinite integral of Dawson’s function [12]. From the fact that ${\tilde{h}}_{1}\left(\xi \right)$ is continuous in ξ and that the expected logarithm is continuous in τ (Proposition 3), and because the expected logarithm of a central $\chi^{2}$-distributed RV of one degree of freedom is −γ−ln(2) (see (A46)), it follows from (A56) that the integration constant *c* in (A86) is

(A87) $$\begin{array}{c} c=-\gamma -2\ln(2). \end{array}$$

(One could also take (A55) and evaluate it for ξ=0 to see that ${\tilde{h}}_{1}\left(0\right)=-\gamma -2\ln\left(2\right)$.)

Comparing ${\tilde{h}}_{1}\left(\cdot \right)$ with $h_{1}\left(\cdot \right)$ now proves that ${\tilde{h}}_{1}\left(\xi \right)=h_{1}\left(\xi \right)$ for all ξ≥0, and thus (54) holds true for n=1.

To prove ${\tilde{h}}_{n}\left(\xi \right)=h_{n}\left(\xi \right)$ for general $n\in N^{\mathrm{odd}}$, we first point out that by comparing (A76) with (52) we see that ${\tilde{h}}_{n}^{\left(1\right)}\left(\xi \right)=h_{n}^{\left(1\right)}\left(\xi \right)$ for all ξ>0 and all $n\in N^{\mathrm{odd}}$. The case ξ=0 follows trivially from (A61).

Next, we use the derivation shown in (A105)–(A108) applied to ${\tilde{h}}_{n}\left(\cdot \right)$ and ${\tilde{h}}_{n}^{\left(1\right)}\left(\cdot \right)$ to show that

(A88) $$\begin{array}{ccc} {\tilde{h}}_{n}\left(\xi \right) & = & {\tilde{h}}_{n-2}\left(\xi \right)+{\tilde{h}}_{n-2}^{\left(1\right)}\left(\xi \right). \end{array}$$

A recursive application of this relation now proves that for all odd n≥3,

(A89) $$\begin{array}{ccc} {\tilde{h}}_{n}\left(\xi \right) & = & {\tilde{h}}_{1}\left(\xi \right)+\sum_{j=1}^{\frac{n-1}{2}}{\tilde{h}}_{2j-1}^{\left(1\right)}\left(\xi \right) \end{array}$$

(also compare with Corollary 7). Plugging (A86) and (A76) into this, and comparing with (45), proves ${\tilde{h}}_{n}\left(\xi \right)=h_{n}\left(\xi \right)$ and thus (54) for all odd *n*.

#### Appendix B.1.2. Negative Integer Moments

To prove the expression of the negative integer moments, fix some ℓ∈N with $\ell \leq \frac{n-1}{2}$. (Note that the result for $\ell >\frac{n-1}{2}$ follows directly from Proposition 1.) We directly focus on ξ>0. We need to evaluate

(A90) $$\begin{array}{c} E\left[U^{-\ell }\right]=\int_{0}^{\infty }u^{-\ell }\cdot \frac{1}{2}{\left(\frac{u}{\tau }\right)}^{\frac{n-2}{4}}\;e^{-\frac{\tau +u}{2}}I_{\frac{n}{2}-1}\left(\sqrt{\tau u}\right)\;du. \end{array}$$

Again using the power series (7), we obtain:

(A91) $$\begin{array}{ccc} E\left[U^{-\ell }\right] & = & \;e^{-\frac{\tau }{2}}\sum_{k=0}^{\infty }\frac{{\left(\frac{\tau }{2}\right)}^{k}}{k!\;\Gamma \left(k+\frac{n}{2}\right)\;2^{k+\frac{n}{2}}}\int_{0}^{\infty }u^{k-\ell +\frac{n}{2}-1}\;e^{-\frac{u}{2}}\;du \end{array}$$

(A92) $$\begin{array}{ccc} & = & \;e^{-\frac{\tau }{2}}\sum_{k=0}^{\infty }\frac{{\left(\frac{\tau }{2}\right)}^{k}}{k!\;\Gamma \left(k+\frac{n}{2}\right)\;2^{k+\frac{n}{2}}}\;2^{\frac{n}{2}+k-\ell }\;\Gamma \left(k-\ell +\frac{n}{2}\right) \end{array}$$

(A93) $$\begin{array}{ccc} & = & 2^{-\ell }\;e^{-\xi }\sum_{k=0}^{\infty }\frac{\Gamma \left(k-\ell +\frac{n}{2}\right)}{k!\;\Gamma \left(k+\frac{n}{2}\right)}\;\xi^{k} \end{array}$$

(A94) $$\begin{array}{ccc} & = & 2^{-\ell }\;e^{-\xi }\sum_{k=0}^{\infty }\frac{\xi^{k}}{k!}\;\frac{1}{\left(k-1+\frac{n}{2}\right)\left(k-2+\frac{n}{2}\right)\cdots \left(k-\ell +\frac{n}{2}\right)}. \end{array}$$

Here, (A92) follows from ([8], Eq. 3.381-4), in (A93) we again use the shorthand (A54), and the last equality (A94) follows because Γ(z+1)=zΓ(z).

Now recall from the equivalence of ${\tilde{h}}_{n}^{\left(1\right)}\left(\xi \right)=h_{n}^{\left(1\right)}\left(\xi \right)$ and from (A61) that

(A95) $$\begin{array}{c} h_{n}^{\left(1\right)}\left(\xi \right)=\;e^{-\xi }\sum_{k=0}^{\infty }\frac{1}{k!\;\left(k+\frac{n}{2}\right)}\;\xi^{k}. \end{array}$$

Thus, the second derivative can be computed to be (uniform convergence of the summation in (A95) can be shown similarly to (A221)–(A228)):

(A96) $$\begin{array}{ccc} h_{n}^{\left(2\right)}\left(\xi \right) & = & -\;e^{-\xi }\sum_{k=0}^{\infty }\frac{1}{k!\;\left(k+\frac{n}{2}\right)}\;\xi^{k}+\;e^{-\xi }\sum_{k=1}^{\infty }\frac{1}{\left(k-1\right)!\;\left(k+\frac{n}{2}\right)}\;\xi^{k-1} \end{array}$$

(A97) $$\begin{array}{ccc} & = & \;e^{-\xi }\sum_{k=0}^{\infty }\frac{\xi^{k}}{k!}\left(\frac{1}{k+1+\frac{n}{2}}-\frac{1}{k+\frac{n}{2}}\right) \end{array}$$

(A98) $$\begin{array}{ccc} & = & \;e^{-\xi }\sum_{k=0}^{\infty }\frac{\xi^{k}}{k!}\;\frac{-1}{\left(k+\frac{n}{2}\right)\left(k+1+\frac{n}{2}\right)}, \end{array}$$

and, in general, the $\ell^{\mathrm{th}}$ derivative is

(A99) $$\begin{array}{ccc} h_{n}^{\left(\ell \right)}\left(\xi \right) & = & \;e^{-\xi }\sum_{k=0}^{\infty }\frac{\xi^{k}}{k!}\;\frac{{\left(-1\right)}^{\ell -1}\left(\ell -1\right)!}{\left(k+\frac{n}{2}\right)\left(k+1+\frac{n}{2}\right)\cdots \left(k+\ell -1+\frac{n}{2}\right)}. \end{array}$$

The claim (56) for $n\in N^{\mathrm{odd}}$ now follows by comparing (A99) with (A94).

### Appendix B.2. Even Degrees of Freedom

Note that all results regarding $U^{\left[n,\tau \right]}$ with $n\in N^{even}$ follow directly from the corresponding result of $V^{\left[m,\lambda \right]}$ using Lemma 1.

#### Appendix B.2.1. Expected Logarithm

The derivation of (55) has been published before in ([1], Lem. 10.1) and ([2], Lem. A.6) (see also [3]). It is similar to the derivation shown in Appendix B.1.1, but easier because in (A51) ψ(·) has an integer argument instead of integer plus $\frac{1}{2}$. This leads to an expression corresponding to (A62) with only integers and thus to a much simpler version of (A63):

(A100) $$\begin{array}{ccc} \sum_{j=0}^{m-1}\frac{{\left(-1\right)}^{j}}{j!\;(\ell -j)!} & = & -\frac{{\left(-1\right)}^{m}}{\ell \;(m-1)!\;(\ell -m)!} \end{array}$$

containing only one term on the right. The change of variables is similar as shown in Figure A1, but again simpler because the sum over the red values in Figure A1 actually equals to zero. We omit further details.

#### Appendix B.2.2. Negative Integer Moments

The derivation of (57) is fully analogous to the derivation shown in Appendix B.1.2. We need to evaluate

(A101) $$\begin{array}{c} E\left[V^{-\ell }\right]=\int_{0}^{\infty }v^{-\ell }\cdot {\left(\frac{v}{\lambda }\right)}^{\frac{m-1}{2}}\;e^{-v-\lambda }I_{m-1}\left(2\sqrt{\lambda v}\right)\;dv. \end{array}$$

Using the power series (7) we obtain from ([8], Eq. 3.351-3) (using that m>ℓ)

(A102) $$\begin{array}{ccc} E\left[V^{-\ell }\right] & = & \lambda^{-\frac{m-1}{2}}\;e^{-\lambda }\sum_{k=0}^{\infty }\frac{1}{k!\;\Gamma (k+m)}\;\lambda^{\frac{2k+m-1}{2}}\int_{0}^{\infty }v^{k+m-1-\ell }\;e^{-v}\;dv \end{array}$$

(A103) $$\begin{array}{ccc} & = & \;e^{-\lambda }\sum_{k=0}^{\infty }\frac{\lambda^{k}}{k!}\;\frac{1}{\left(k+m-1)\cdots (k+m-\ell \right)}. \end{array}$$

Using the corresponding expression of the $\ell^{\mathrm{th}}$ derivative of $g_{m}\left(\cdot \right)$, which is derived similarly to (A98),

(A104) $$\begin{array}{c} g_{m}^{\left(\ell \right)}\left(\xi \right)=\;e^{-\xi }\sum_{k=0}^{\infty }\frac{\xi^{k}}{k!}\;\frac{{\left(-1\right)}^{\ell -1}\left(\ell -1\right)!}{\left(k+m)\cdots (k+m+\ell -1\right)}, \end{array}$$

we obtain the claimed result.

## Appendix C. Proofs of Section 5

### Appendix C.1. Proof of Theorems 3 and 5

We start with $h_{n}\left(\cdot \right)$. To prove (76a), we use (A55) to write

(A105) $$\begin{array}{ccc} h_{n+2}\left(\xi \right)-h_{n}\left(\xi \right) & = & \;e^{-\xi }\sum_{k=0}^{\infty }\frac{\xi^{k}}{k!}\sum_{j=1}^{\frac{n+1}{2}+k}\frac{1}{j-\frac{1}{2}}-\;e^{-\xi }\sum_{k=0}^{\infty }\frac{\xi^{k}}{k!}\sum_{j=1}^{\frac{n-1}{2}+k}\frac{1}{j-\frac{1}{2}} \end{array}$$

(A106) $$\begin{array}{ccc} & = & \;e^{-\xi }\sum_{k=0}^{\infty }\frac{\xi^{k}}{k!}\;\frac{1}{\frac{n+1}{2}+k-\frac{1}{2}} \end{array}$$

(A107) $$\begin{array}{ccc} & = & \;e^{-\xi }\sum_{k=0}^{\infty }\frac{\xi^{k}}{k!}\;\frac{1}{\frac{n}{2}+k} \end{array}$$

(A108) $$\begin{array}{ccc} & = & h_{n}^{\left(1\right)}\left(\xi \right), \end{array}$$

where the last equality follows from (A95).

To prove (76b), we use (A99) to write

$$\begin{array}{ccc} h_{n+2}^{\left(\ell \right)}\left(\xi \right)-h_{n}^{\left(\ell \right)}\left(\xi \right) & = & \;e^{-\xi }\sum_{k=0}^{\infty }\frac{\xi^{k}}{k!}\;\frac{{\left(-1\right)}^{\ell -1}\left(\ell -1\right)!}{\left(k+\frac{n}{2}+1\right)\left(k+2+\frac{n}{2}\right)\cdots \left(k+\ell +\frac{n}{2}\right)} \end{array}$$

(A109) $$\begin{array}{ccc} & & -\;e^{-\xi }\sum_{k=0}^{\infty }\frac{\xi^{k}}{k!}\;\frac{{\left(-1\right)}^{\ell -1}\left(\ell -1\right)!}{\left(k+\frac{n}{2}\right)\left(k+1+\frac{n}{2}\right)\cdots \left(k+\ell -1+\frac{n}{2}\right)} \end{array}$$

(A110) $$\begin{array}{ccc} & = & {\left(-1\right)}^{\ell -1}\left(\ell -1\right)!\;e^{-\xi }\sum_{k=0}^{\infty }\frac{\xi^{k}}{k!}\;\frac{k+\frac{n}{2}-k-\ell -\frac{n}{2}}{\left(k+\frac{n}{2}\right)\left(k+1+\frac{n}{2}\right)\cdots \left(k+\ell +\frac{n}{2}\right)} \end{array}$$

(A111) $$\begin{array}{ccc} & = & {\left(-1\right)}^{\ell }\ell !\;e^{-\xi }\sum_{k=0}^{\infty }\frac{\xi^{k}}{k!}\;\frac{1}{\left(k+\frac{n}{2}\right)\left(k+1+\frac{n}{2}\right)\cdots \left(k+\ell +\frac{n}{2}\right)} \end{array}$$

(A112) $$\begin{array}{ccc} & = & h_{n}^{\left(\ell +1\right)}\left(\xi \right). \end{array}$$

The derivations for $g_{m}\left(\cdot \right)$ are fully analogous. In particular, we can use the equivalent of (A55), i.e.,

(A113) $$\begin{array}{c} g_{m}\left(\xi \right)=-\gamma +\;e^{-\xi }\sum_{k=0}^{\infty }\frac{\xi^{k}}{k!}\sum_{j=1}^{k+m-1}\frac{1}{j}, \end{array}$$

to rewrite the corresponding version of (A105)–(A108). For the interested reader we show a different, slightly more cumbersome proof that directly relies on the definition of $g_{m}\left(\xi \right)$ and $g_{m}^{\left(1\right)}\left(\xi \right)$ in (35) and (39), respectively:

(A114) $$\begin{array}{cc} g_{m}\left(\xi \right)+g_{m}^{\left(1\right)}\left(\xi \right) & \\ = & \ln\left(\xi \right)-\mathrm{Ei}\left(-\xi \right)+\sum_{j=1}^{m-1}{\left(-1\right)}^{j}\;e^{-\xi }\left(j-1\right)!{\left(\frac{1}{\xi }\right)}^{j}-\sum_{j=1}^{m-1}{\left(-1\right)}^{j}\frac{(m-1)!}{j(m-1-j)!}{\left(\frac{1}{\xi }\right)}^{j}\\ + & \;\frac{{\left(-1\right)}^{m}\left(m-1\right)!}{\xi^{m}}\;e^{-\xi }-\sum_{i=0}^{m-1}{\left(-1\right)}^{i+m}\frac{(m-1)!}{i!}\xi^{i-m} \end{array}$$

(A115) $$\begin{array}{ccc} & = & \ln\left(\xi \right)-\mathrm{Ei}\left(-\xi \right)+\sum_{j=1}^{m}{\left(-1\right)}^{j}\;e^{-\xi }\left(j-1\right)!{\left(\frac{1}{\xi }\right)}^{j}-\sum_{j=1}^{m-1}{\left(-1\right)}^{j}\frac{(m-1)!}{j(m-1-j)!}{\left(\frac{1}{\xi }\right)}^{j}\\ & & -\sum_{j=1}^{m}\underset{={\left(-1\right)}^{j}}{\underbrace{{\left(-1\right)}^{-j+2m}}}\frac{(m-1)!}{(m-j)!}\;\xi^{-j} \end{array}$$

(A116) $$\begin{array}{ccc} & = & \ln\left(\xi \right)-\mathrm{Ei}\left(-\xi \right)+\sum_{j=1}^{m}{\left(-1\right)}^{j}\;e^{-\xi }\left(j-1\right)!{\left(\frac{1}{\xi }\right)}^{j}\\ & & -\sum_{j=1}^{m-1}{\left(-1\right)}^{j}\left(m-1\right)!\underset{=\frac{m}{j(m-j)!}}{\underbrace{\left(\frac{1}{j(m-1-j)!}+\frac{1}{(m-j)!}\right)}}{\left(\frac{1}{\xi }\right)}^{j}-{\left(-1\right)}^{m}\frac{(m-1)!}{0!}{\left(\frac{1}{\xi }\right)}^{m} \end{array}$$

(A117) $$\begin{array}{ccc} & = & \ln\left(\xi \right)-\mathrm{Ei}\left(-\xi \right)+\sum_{j=1}^{m}{\left(-1\right)}^{j}\;e^{-\xi }\left(j-1\right)!{\left(\frac{1}{\xi }\right)}^{j}-\sum_{j=1}^{m-1}{\left(-1\right)}^{j}\left(m-1\right)!\frac{m}{j(m-j)!}{\left(\frac{1}{\xi }\right)}^{j}\\ & & -\;{\left(-1\right)}^{m}\frac{m!}{m\;0!}{\left(\frac{1}{\xi }\right)}^{m} \end{array}$$

(A118) $$\begin{array}{ccc} & = & \ln\left(\xi \right)-\mathrm{Ei}\left(-\xi \right)+\sum_{j=1}^{m}{\left(-1\right)}^{j}\;e^{-\xi }\left(j-1\right)!{\left(\frac{1}{\xi }\right)}^{j}-\sum_{j=1}^{m}{\left(-1\right)}^{j}\frac{m!}{j(m-j)!}{\left(\frac{1}{\xi }\right)}^{j} \end{array}$$

(A119) $$\begin{array}{ccc} & = & g_{m+1}\left(\xi \right). \end{array}$$

Here, the first equality follows from the definitions given in (35) and (39); in the subsequent equality we combine the second last term with the first sum and reorder the last summation by introducing a new counter-variable j=m−i; the subsequent three equalities follow from arithmetic rearrangements; and the final equality follows again from definition (35). This proves (63a).

To prove (63b), we use (A104) to write

$$\begin{array}{c} g_{m+1}^{\left(\ell \right)}\left(\xi \right)-g_{m}^{\left(\ell \right)}\left(\xi \right) \end{array}$$

(A120) $$\begin{array}{ccc} \; & = & \;e^{-\xi }\sum_{k=1}^{\infty }\frac{\xi^{k}}{k!}{\left(-1\right)}^{\ell -1}\left(\ell -1\right)!\left(\frac{1}{\left(k+m+1)\cdots (k+m+\ell \right)}-\frac{1}{\left(k+m)\cdots (k+m+\ell -1\right)}\right) \end{array}$$

(A121) $$\begin{array}{ccc} & = & \;e^{-\xi }\sum_{k=1}^{\infty }\frac{\xi^{k}}{k!}{\left(-1\right)}^{\ell -1}\left(\ell -1\right)!\;\frac{k+m-(k+m+\ell )}{\left(k+m)\cdots (k+m+\ell \right)} \end{array}$$

(A122) $$\begin{array}{ccc} & = & \;e^{-\xi }\sum_{k=1}^{\infty }\frac{\xi^{k}}{k!}{\left(-1\right)}^{\ell }\ell !\;\frac{1}{\left(k+m)\cdots (k+m+\ell \right)} \end{array}$$

(A123) $$\begin{array}{ccc} & = & g_{m}^{\left(\ell +1\right)}\left(\xi \right). \end{array}$$

### Appendix C.2. Proof of Theorems 4 and 6

Using (60) we have

(A124) $$\begin{array}{ccc} \frac{1}{\xi }-\frac{m}{\xi }g_{m}^{\left(1\right)}\left(\xi \right) & = & \frac{1}{\xi }\;e^{-\xi }\cdot \;e^{\xi }-\frac{m}{\xi }\;e^{-\xi }\sum_{k=0}^{\infty }\frac{1}{k!}\cdot \frac{1}{k+m}\cdot \xi^{k} \end{array}$$

(A125) $$\begin{array}{ccc} & = & \frac{1}{\xi }\;e^{-\xi }\sum_{k=0}^{\infty }\frac{1}{k!}\cdot \xi^{k}-\frac{1}{\xi }\;e^{-\xi }\sum_{k=0}^{\infty }\frac{1}{k!}\cdot \frac{m}{k+m}\cdot \xi^{k} \end{array}$$

(A126) $$\begin{array}{ccc} & = & \frac{1}{\xi }\;e^{-\xi }\sum_{k=0}^{\infty }\frac{1}{k!}\left(1-\frac{m}{k+m}\right)\xi^{k} \end{array}$$

(A127) $$\begin{array}{ccc} & = & \;e^{-\xi }\sum_{k=0}^{\infty }\frac{1}{k!}\cdot \frac{k}{k+m}\cdot \xi^{k-1} \end{array}$$

(A128) $$\begin{array}{ccc} & = & \;e^{-\xi }\sum_{k=1}^{\infty }\frac{1}{(k-1)!}\cdot \frac{1}{k+m}\cdot \xi^{k-1} \end{array}$$

(A129) $$\begin{array}{ccc} & = & \;e^{-\xi }\sum_{k=0}^{\infty }\frac{1}{k!}\cdot \frac{1}{k+m+1}\cdot \xi^{k} \end{array}$$

(A130) $$\begin{array}{ccc} & = & g_{m+1}^{\left(1\right)}\left(\xi \right). \end{array}$$

Here, the first equality follows from (60); in the subsequent equality we use the series expansion of $\;e^{\xi }$; the subsequent two equalities follow from algebraic rearrangements; in the next equality we note that for k=0 the terms in the sum are equal to zero; the second last equality then follows from renumbering the terms; and the last equality follows again from (60). This proves (73).

The derivation of (83) is completely analogous, but where *m* is replaced by $\frac{n}{2}$ in most places and where we rely on (A61) instead of (60):

(A131) $$\begin{array}{ccc} \frac{1}{\xi }-\frac{\frac{n}{2}}{\xi }\;h_{n}^{\left(1\right)}\left(\xi \right) & = & \frac{1}{\xi }\;e^{-\xi }\sum_{k=0}^{\infty }\frac{1}{k!}\cdot \xi^{k}-\frac{1}{\xi }\;e^{-\xi }\sum_{k=0}^{\infty }\frac{1}{k!}\cdot \frac{\frac{n}{2}}{k+\frac{n}{2}}\cdot \xi^{k} \end{array}$$

(A132) $$\begin{array}{ccc} & = & \frac{1}{\xi }\;e^{-\xi }\sum_{k=0}^{\infty }\frac{1}{k!}\left(1-\frac{\frac{n}{2}}{k+\frac{n}{2}}\right)\xi^{k} \end{array}$$

(A133) $$\begin{array}{ccc} & = & \;e^{-\xi }\sum_{k=0}^{\infty }\frac{1}{k!}\cdot \frac{k}{k+\frac{n}{2}}\cdot \xi^{k-1} \end{array}$$

(A134) $$\begin{array}{ccc} & = & \;e^{-\xi }\sum_{k=1}^{\infty }\frac{1}{(k-1)!}\cdot \frac{1}{k+\frac{n}{2}}\cdot \xi^{k-1} \end{array}$$

(A135) $$\begin{array}{ccc} & = & \;e^{-\xi }\sum_{k=0}^{\infty }\frac{1}{k!}\cdot \frac{1}{k+\frac{n}{2}+1}\cdot \xi^{k} \end{array}$$

(A136) $$\begin{array}{ccc} & = & h_{n+2}^{\left(1\right)}\left(\xi \right). \end{array}$$

## Appendix D. Proofs of Section 6

### Appendix D.1. Proof of Theorems 7 and 8

We start with the proof of Theorem 8, because the derivation of the bounds on $g_{m}\left(\cdot \right)$ depend strongly on the bounds on $g_{m}^{\left(1\right)}\left(\cdot \right)$.

We start with the observation that (105) holds with equality for ξ=0. Moreover, we notice that the bound is asymptotically tight, too:

(A137) $$\begin{array}{ccc} \lim_{\xi ↑\infty }g_{m}^{\left(1\right)}\left(\xi \right) & = & 0, \end{array}$$

(A138) $$\begin{array}{ccc} \lim_{\xi ↑\infty }\frac{1}{\xi +m} & = & 0 \end{array}$$

(the first equality follows directly from (39)). Since additionally both functions $\xi \mapsto g_{m}^{\left(1\right)}\left(\xi \right)$ and $\xi \mapsto \frac{1}{\xi +m}$ are monotonically strictly decreasing and strictly convex, they cannot cross. So, it suffices to find some ξ for which (105) is satisfied. We pick ξ=1 and check:

(A139) $$\begin{array}{ccc} g_{m}^{\left(1\right)}\left(1\right) & = & {\left(-1\right)}^{m}\left(m-1\right)!\left(\;e^{-1}-\sum_{j=0}^{m-1}\frac{{\left(-1\right)}^{j}}{j!}\right) \end{array}$$

(A140) $$\begin{array}{ccc} & = & {\left(-1\right)}^{m}\left(m-1\right)!\left(\sum_{j=0}^{\infty }\frac{{\left(-1\right)}^{j}}{j!}-\sum_{j=0}^{m-1}\frac{{\left(-1\right)}^{j}}{j!}\right) \end{array}$$

(A141) $$\begin{array}{ccc} & = & {\left(-1\right)}^{m}\left(m-1\right)!\sum_{j=m}^{\infty }\frac{{\left(-1\right)}^{j}}{j!} \end{array}$$

(A142) $$\begin{array}{ccc} & = & \sum_{j=m,m+2,m+4,\ldots }\left(\frac{{\left(-1\right)}^{j+m}\;\left(m-1\right)!}{j!}+\frac{{\left(-1\right)}^{j+1+m}\;\left(m-1\right)!}{(j+1)!}\right) \end{array}$$

(A143) $$\begin{array}{ccc} & = & \sum_{j=m,m+2,m+4,\ldots }\left(\frac{(m-1)!}{j!}-\frac{(m-1)!}{(j+1)!}\right) \end{array}$$

(A144) $$\begin{array}{ccc} & = & \sum_{j=m,m+2,m+4,\ldots }\frac{(m-1)!\;j}{(j+1)!} \end{array}$$

(A145) $$\begin{array}{ccc} & > & \frac{(m-1)!\;m}{(m+1)!} \end{array}$$

(A146) $$\begin{array}{ccc} & = & \frac{1}{m+1}. \end{array}$$

Here, (A141) follows from the series expansion of the exponential function; in (A142) we split the sum into two sums over the even and odd values of *j*; (A143) holds because j+m is even and j+m+1 is odd; and the inequality (A145) follows from dropping all terms in the sum (they are all positive!) apart from the first.

Next, we turn to (106). From Theorem 4 we have for any m∈N,

(A147) $$\begin{array}{ccc} g_{m}^{\left(1\right)}\left(\xi \right) & = & \frac{1-\xi \;g_{m+1}^{\left(1\right)}\left(\xi \right)}{m} \end{array}$$

(A148) $$\begin{array}{ccc} & \leq & \frac{1-\xi \;\frac{1}{\xi +m+1}}{m} \end{array}$$

(A149) $$\begin{array}{ccc} & = & \frac{m+1}{m(\xi +m+1)}, \end{array}$$

where the inequality follows from (105).

To derive (107), we first look at the case m≥2 and consider the difference between the expression of the upper bound and $g_{m}^{\left(1\right)}\left(\xi \right)$:

(A150) $$\begin{array}{ccc} \frac{1}{\xi +m-1}-g_{m}^{\left(1\right)}\left(\xi \right) & = & \frac{1}{\xi +m-1}-\frac{1}{\xi }+\frac{m-1}{\xi }g_{m-1}^{\left(1\right)}\left(\xi \right) \end{array}$$

(A151) $$\begin{array}{ccc} & \geq & \frac{1}{\xi +m-1}-\frac{1}{\xi }+\frac{m-1}{\xi }\frac{1}{\xi +m-1} \end{array}$$

(A152) $$\begin{array}{ccc} & = & 0, \end{array}$$

where the first equality follows from Theorem 4 and the subsequent inequality from the lower bound (105). For m=1 and ξ=0, (107) holds trivially, so it remains to show the case m=1 and ξ>0. This follows directly from (39):

(A153) $$\begin{array}{ccc} g_{1}^{\left(1\right)}\left(\xi \right) & = & \frac{1-\;e^{-\xi }}{\xi }\leq \frac{1}{\xi }. \end{array}$$

We next address the claims in Theorem 7.

The upper bound (101) has been proven before in ([15], App. B) and is based on Jensen’s inequality:

(A154) $$\begin{array}{ccc} g_{m}\left(\lambda \right) & = & E\left[\ln\left(V^{\left[m,\lambda \right]}\right)\right] \end{array}$$

(A155) $$\begin{array}{ccc} & \leq & \ln\left(E\left[V^{\left[m,\lambda \right]}\right]\right) \end{array}$$

(A156) $$\begin{array}{ccc} & = & \ln(m+\lambda ). \end{array}$$

The lower bound (99) follows from a slightly more complicated argument: Note that both $\xi \mapsto g_{m}\left(\xi \right)$ and ξ↦ln(ξ+m−1) are monotonically strictly increasing and strictly concave functions (see Proposition 6). Hence, they can cross at most twice. Asymptotically as ξ↑∞ the two functions coincide, i.e., this corresponds to one of these “crossings.” (This can be seen directly from (A156).) So, they can only cross at most once more for finite ξ. For ξ=0, we have

(A157) $$\begin{array}{c} g_{m}\left(0\right)=\psi \left(m\right)>\ln\left(m-1\right) \end{array}$$

for all m∈N (where for m=1 we take ln(0)=−∞), see, e.g., ([16], Eq. (94)).

By contradiction, let us assume for the moment that there is another crossing at a finite value. At that value, the slope of ξ↦ln(ξ+m−1) is larger than the slope of $\xi \mapsto g_{m}\left(\xi \right)$. Since asymptotically the two function coincide again, there must exist some value $\xi_{0}$ such that for $\xi >\xi_{0}$ the slope of ξ↦ln(ξ+m−1) is strictly smaller than the slope of $\xi \mapsto g_{m}\left(\xi \right)$. We know from (107), however, that

(A158) $$\begin{array}{c} \frac{\partial }{\partial \xi }\ln\left(\xi +m-1\right)=\frac{1}{\xi +m-1}\geq g_{m}^{\left(1\right)}\left(\xi \right),\;\forall \;\xi \geq 0, \end{array}$$

which leads to a contradiction. Thus, there cannot be another crossing and ln(ξ+m−1) must be strictly smaller that $g_{m}\left(\xi \right)$ for all ξ≥0.

The lower and upper bounds (100) and (102) rely on the fundamental theorem of calculus:

(A159) $$\begin{array}{ccc} g_{m}\left(\xi \right)-g_{m}\left(0\right) & = & \int_{0}^{\xi }g_{m}^{\left(1\right)}\left(t\right)\;dt \end{array}$$

(A160) $$\begin{array}{ccc} & \geq & \int_{0}^{\xi }\frac{1}{t+m}\;dt \end{array}$$

(A161) $$\begin{array}{ccc} & = & {\left[\ln(t+m)\right]}_{0}^{\xi } \end{array}$$

(A162) $$\begin{array}{ccc} & = & \ln(\xi +m)-\ln(m), \end{array}$$

where the inequality follows from (105). Thus,

(A163) $$\begin{array}{ccc} g_{m}\left(\xi \right) & \geq & \ln\left(\xi +m\right)-\ln\left(m\right)+g_{m}\left(0\right) \end{array}$$

(A164) $$\begin{array}{ccc} & = & \ln(\xi +m)-\ln(m)+\psi (m). \end{array}$$

Similarly,

(A165) $$\begin{array}{ccc} g_{m}\left(\xi \right) & = & \int_{0}^{\xi }g_{m}^{\left(1\right)}\left(t\right)\;dt+g_{m}\left(0\right) \end{array}$$

(A166) $$\begin{array}{ccc} & \leq & \int_{0}^{\xi }\frac{m+1}{m(t+m+1)}\;dt+\psi \left(m\right) \end{array}$$

(A167) $$\begin{array}{ccc} & = & \frac{m+1}{m}\ln\left(\xi +m+1\right)-\frac{m+1}{m}\ln\left(m+1\right)+\psi \left(m\right), \end{array}$$

where the inequality follows from (106).

### Appendix D.2. Proof of Theorems 9 and 10

We start with the proof of Theorem 10, because the derivation of the bounds on $h_{n}\left(\cdot \right)$ depend strongly on the bounds on $h_{n}^{\left(1\right)}\left(\cdot \right)$.

We start with the observation that (117) holds with equality for ξ=0. Moreover, we notice that the bound is asymptotically tight, too:

(A168) $$\begin{array}{ccc} \lim_{\xi ↑\infty }h_{n}^{\left(1\right)}\left(\xi \right) & = & 0, \end{array}$$

(A169) $$\begin{array}{ccc} \lim_{\xi ↑\infty }\frac{1}{\xi +\frac{n}{2}} & = & 0 \end{array}$$

(the first equality follows directly from (52)). Since additionally both functions $\xi \mapsto h_{n}^{\left(1\right)}\left(\xi \right)$ and $\xi \mapsto \frac{1}{\xi +\frac{n}{2}}$ are monotonically strictly decreasing and strictly convex, they cannot cross. So, it suffices to find some ξ for which (117) is satisfied. We pick ξ=1 and check:

(A170) $$\begin{array}{ccc} h_{n}^{\left(1\right)}\left(1\right) & = & {\left(-1\right)}^{\frac{n-1}{2}}\Gamma \left(\frac{n}{2}\right)\left(\;e^{-1}\mathrm{erfi}\left(1\right)+\sum_{j=1}^{\frac{n-1}{2}}\frac{{\left(-1\right)}^{j}}{\Gamma \left(j+\frac{1}{2}\right)}\right) \end{array}$$

(A171) $$\begin{array}{ccc} & = & {\left(-1\right)}^{\frac{n-1}{2}}\Gamma \left(\frac{n}{2}\right)\left(\sum_{k=0}^{\infty }\frac{{\left(-1\right)}^{k}}{\Gamma \left(k+\frac{3}{2}\right)}-\sum_{k=0}^{\frac{n-3}{2}}\frac{{\left(-1\right)}^{k}}{\Gamma \left(k+\frac{3}{2}\right)}\right) \end{array}$$

(A172) $$\begin{array}{ccc} & = & {\left(-1\right)}^{\frac{n-1}{2}}\Gamma \left(\frac{n}{2}\right)\sum_{k=\frac{n-1}{2}}^{\infty }\frac{{\left(-1\right)}^{k}}{\Gamma \left(k+\frac{3}{2}\right)} \end{array}$$

(A173) $$\begin{array}{ccc} & = & \sum_{k=\frac{n-1}{2}}^{\infty }\frac{{\left(-1\right)}^{k+\frac{n-1}{2}}\;\Gamma \left(\frac{n}{2}\right)}{\Gamma \left(k+\frac{3}{2}\right)} \end{array}$$

(A174) $$\begin{array}{ccc} & = & \sum_{k=\frac{n-1}{2},\frac{n-1}{2}+2,\frac{n-1}{2}+4,\ldots }\left(\frac{\Gamma \left(\frac{n}{2}\right)}{\Gamma \left(k+\frac{3}{2}\right)}-\frac{\Gamma \left(\frac{n}{2}\right)}{\Gamma \left(k+\frac{5}{2}\right)}\right) \end{array}$$

(A175) $$\begin{array}{ccc} & = & \sum_{k=\frac{n-1}{2},\frac{n-1}{2}+2,\frac{n-1}{2}+4,\ldots }\frac{\left(k+\frac{3}{2}-1\right)\;\Gamma \left(\frac{n}{2}\right)}{\left(k+\frac{3}{2}\right)\;\Gamma \left(k+\frac{3}{2}\right)} \end{array}$$

(A176) $$\begin{array}{ccc} & > & \frac{\left(\frac{n-1}{2}+\frac{1}{2}\right)\;\Gamma \left(\frac{n}{2}\right)}{\left(\frac{n-1}{2}+\frac{3}{2}\right)\;\Gamma \left(\frac{n-1}{2}+\frac{3}{2}\right)} \end{array}$$

(A177) $$\begin{array}{ccc} & = & \frac{\frac{n}{2}\;\Gamma \left(\frac{n}{2}\right)}{\left(\frac{n}{2}+1\right)\Gamma \left(\frac{n}{2}+1\right)} \end{array}$$

(A178) $$\begin{array}{ccc} & = & \frac{\Gamma \left(\frac{n}{2}+1\right)}{\left(\frac{n}{2}+1\right)\Gamma \left(\frac{n}{2}+1\right)} \end{array}$$

(A179) $$\begin{array}{ccc} & = & \frac{1}{\frac{n}{2}+1}. \end{array}$$

Here, (A171) follows from the series expansion of Dawson’s function [12]

(A180) $$\begin{array}{c} \frac{\sqrt{\pi }}{2}\;e^{-\xi }\mathrm{erfi}\left(\sqrt{\xi }\right)=D\left(\sqrt{\xi }\right)=\frac{\sqrt{\pi }}{2}\sum_{k=0}^{\infty }\frac{{\left(-1\right)}^{k}}{\Gamma \left(k+\frac{3}{2}\right)}\;\xi^{k+\frac{1}{2}}, \end{array}$$

and from a substitution k=j−1; in (A174) we split the sum into two sums over the even and odd values of *k*; in (A175) we combine the terms using the relation zΓ(z)=Γ(z+1); the inequality (A176) follows from dropping all terms in the sum (they are all positive!) apart from the first; and (A178) follows again from zΓ(z)=Γ(z+1).

Next we turn to (118). From Theorem 6 we have for any $n\in N^{\mathrm{odd}}$,

(A181) $$\begin{array}{ccc} h_{n}^{\left(1\right)}\left(\xi \right) & = & \frac{1-\xi \;h_{n+2}^{\left(1\right)}\left(\xi \right)}{\frac{n}{2}} \end{array}$$

(A182) $$\begin{array}{ccc} & \leq & \frac{1-\xi \;\frac{1}{\xi +\frac{n+2}{2}}}{\frac{n}{2}} \end{array}$$

(A183) $$\begin{array}{ccc} & = & \frac{n+2}{n(\xi +\frac{n}{2}+1)}, \end{array}$$

where the inequality follows from (117).

To derive (119a), we consider the difference between the expression of the upper bound and $h_{n}^{\left(1\right)}\left(\xi \right)$:

(A184) $$\begin{array}{ccc} \frac{1}{\xi +\frac{n}{2}-1}-h_{n}^{\left(1\right)}\left(\xi \right) & = & \frac{1}{\xi +\frac{n}{2}-1}-\frac{1}{\xi }+\frac{n-2}{2\xi }h_{n-2}^{\left(1\right)}\left(\xi \right) \end{array}$$

(A185) $$\begin{array}{ccc} & \geq & \frac{1}{\xi +\frac{n}{2}-1}-\frac{1}{\xi }+\frac{n-2}{2\xi }\frac{1}{\xi +\frac{n-2}{2}} \end{array}$$

(A186) $$\begin{array}{ccc} & = & 0, \end{array}$$

where the first equality follows from Theorem 6 (with n≥3) and the subsequent inequality from the lower bound (117). For a derivation of (119b), we start with (A61):

(A187) $$\begin{array}{ccc} h_{1}^{\left(1\right)}\left(\xi \right) & = & \;e^{-\xi }\sum_{k=0}^{\infty }\frac{1}{k!\left(k+\frac{1}{2}\right)}\cdot \xi^{k} \end{array}$$

(A188) $$\begin{array}{ccc} & = & \frac{2}{\xi }\;e^{-\xi }\sum_{k=0}^{\infty }\frac{1}{k!(2k+1)}\cdot \xi^{k+1} \end{array}$$

(A189) $$\begin{array}{ccc} & \leq & \frac{2}{\xi }\;e^{-\xi }\sum_{k=0}^{\infty }\frac{1}{k!(k+1)}\cdot \xi^{k+1} \end{array}$$

(A190) $$\begin{array}{ccc} & = & \frac{2}{\xi }\;e^{-\xi }\sum_{k=0}^{\infty }\frac{1}{(k+1)!}\cdot \xi^{k+1} \end{array}$$

(A191) $$\begin{array}{ccc} & = & \frac{2}{\xi }\;e^{-\xi }\sum_{k=1}^{\infty }\frac{1}{k!}\cdot \xi^{k} \end{array}$$

(A192) $$\begin{array}{ccc} & = & \frac{2}{\xi }\;e^{-\xi }\left(\sum_{k=0}^{\infty }\frac{1}{k!}\cdot \xi^{k}-1\right) \end{array}$$

(A193) $$\begin{array}{ccc} & = & \frac{2}{\xi }\;e^{-\xi }\left(\;e^{\xi }-1\right) \end{array}$$

(A194) $$\begin{array}{ccc} & = & \frac{2}{\xi }\left(1-\;e^{-\xi }\right). \end{array}$$

Here, the inequality holds because 2k+1≥k+1; and in (A193) we again rely on the series expansion of the exponential function. The weaker version of (119b) follows directly from this because $\;e^{-\xi }\geq 0$. This finishes the proof of Theorem 10.

We next address the claims in Theorem 9.

The upper bound (112) is based on Jensen’s inequality:

(A195) $$\begin{array}{ccc} \ln\left(2\right)+h_{n}\left(\frac{\tau }{2}\right) & = & E\left[\ln\left(U^{\left[n,\tau \right]}\right)\right] \end{array}$$

(A196) $$\begin{array}{ccc} & \leq & \ln\left(E\left[U^{\left[n,\tau \right]}\right]\right) \end{array}$$

(A197) $$\begin{array}{ccc} & = & \ln(n+\tau ). \end{array}$$

Thus,

(A198) $$\begin{array}{ccc} h_{n}\left(\xi \right) & \leq & \ln\left(n+2\xi \right)-\ln\left(2\right)=\ln\left(\frac{n}{2}+\xi \right). \end{array}$$

The lower bound (110a) follows from a slightly more complicated argument. Note that for n≥3, both $\xi \mapsto h_{n}\left(\xi \right)$ and $\xi \mapsto \ln\left(\xi +\frac{n}{2}-1\right)$ are monotonically strictly increasing and strictly concave functions (see Proposition 10). Hence, they can cross at most twice. Asymptotically as ξ↑∞ the two functions coincide (this can be seen directly from (A198)), i.e., this corresponds to one of these “crossings.” So, they can only cross at most once more for finite ξ. For ξ=0, we have

(A199) $$\begin{array}{c} h_{n}\left(0\right)=\psi \left(\frac{n}{2}\right)>\ln\left(\frac{n}{2}-1\right) \end{array}$$

for all n≥3, see, e.g., ([16], Eq. (94)). By contradiction, let us assume for the moment that there is another crossing at a finite value. At that value, the slope of $\xi \mapsto \ln\left(\xi +\frac{n}{2}-1\right)$ is larger than the slope of $\xi \mapsto h_{n}\left(\xi \right)$. Since asymptotically the two function coincide again, there must exist some value $\xi_{0}$ such that for $\xi >\xi_{0}$ the slope of $\xi \mapsto \ln\left(\xi +\frac{n}{2}-1\right)$ is strictly smaller than the slope of $\xi \mapsto h_{n}\left(\xi \right)$. We know from (199a), however, that

(A200) $$\begin{array}{c} \frac{\partial }{\partial \xi }\ln\left(\xi +\frac{n}{2}-1\right)=\frac{1}{\xi +\frac{n}{2}-1}\geq h_{n}^{\left(1\right)}\left(\xi \right),\;\forall \;\xi \geq 0, \end{array}$$

which leads to a contradiction. Thus, there cannot be another crossing and $\ln\left(\xi +\frac{n}{2}-1\right)$ must be strictly smaller that $h_{n}\left(\xi \right)$ for all ξ≥0 and n≥3.

To derive the lower bound (110b), we use (76a) in Theorem 5 and apply (110a) and (119b):

(A201) $$\begin{array}{ccc} h_{1}\left(\xi \right) & = & h_{3}\left(\xi \right)-h_{1}^{\left(1\right)}\left(\xi \right) \end{array}$$

(A202) $$\begin{array}{ccc} & \geq & \ln\left(\xi +\frac{1}{2}\right)-\frac{2}{\xi }\left(1-\;e^{-\xi }\right). \end{array}$$

The upper and lower bounds (111) and (113) rely on the fundamental theorem of calculus:

(A203) $$\begin{array}{ccc} h_{n}\left(\xi \right)-h_{n}\left(0\right) & = & \int_{0}^{\xi }h_{n}^{\left(1\right)}\left(t\right)\;dt \end{array}$$

(A204) $$\begin{array}{ccc} & \geq & \int_{0}^{\xi }\frac{1}{t+\frac{n}{2}}\;dt \end{array}$$

(A205) $$\begin{array}{ccc} & = & {\left[\ln\left(t+\frac{n}{2}\right)\right]}_{0}^{\xi } \end{array}$$

(A206) $$\begin{array}{ccc} & = & \ln\left(\xi +\frac{n}{2}\right)-\ln\left(\frac{n}{2}\right), \end{array}$$

where the inequality follows from (117). Thus,

(A207) $$\begin{array}{ccc} h_{n}\left(\xi \right) & \geq & \ln\left(\xi +\frac{n}{2}\right)-\ln\left(\frac{n}{2}\right)+h_{n}\left(0\right) \end{array}$$

(A208) $$\begin{array}{ccc} & = & \ln\left(\xi +\frac{n}{2}\right)-\ln\left(\frac{n}{2}\right)+\psi \left(\frac{n}{2}\right). \end{array}$$

Similarly,

(A209) $$\begin{array}{ccc} h_{n}\left(\xi \right) & = & \int_{0}^{\xi }h_{n}^{\left(1\right)}\left(t\right)\;dt+h_{n}\left(0\right) \end{array}$$

(A210) $$\begin{array}{ccc} & \leq & \int_{0}^{\xi }\frac{n+2}{n\left(t+\frac{n}{2}+1\right)}\;dt+\psi \left(\frac{n}{2}\right) \end{array}$$

(A211) $$\begin{array}{ccc} & = & \frac{n+2}{n}\ln\left(\xi +\frac{n}{2}+1\right)-\frac{n+2}{n}\ln\left(\frac{n}{2}+1\right)+\psi \left(\frac{n}{2}\right), \end{array}$$

where the inequality follows from (118).

## Appendix E. Uniform Convergence

In the following we will prove uniform convergence using Weierstrass’ M-test ([13], Sec. 8.11): An infinite sum $\sum_{k=0}^{\infty }f_{k}\left(x\right)$ converges uniformly for all x∈X⊆R, if we can find constants $M_{k}$ that do not depend on *x*, that satisfy

(A212) $$\begin{array}{c} |f_{k}\left(x\right)|\leq M_{k},\;\forall \;x\in X, \end{array}$$

and whose sum converges:

(A213) $$\begin{array}{c} \sum_{k=0}^{\infty }M_{k}\;\mathrm{is}\;\mathrm{converging}. \end{array}$$

The condition (A213) can be confirmed by d’Alembert’s ratio test [17]: if

(A214) $$\begin{array}{c} \lim_{k\to \infty }\frac{M_{k+1}}{M_{k}}<1 \end{array}$$

then the sum in (A213) indeed converges.

### Appendix E.1. Uniform Convergence of *(A49)*

We assume ξ≥0 and note that $\xi \mapsto \xi^{k+\frac{n}{2}-1}\;e^{-\xi }$ has its maximum for $\xi =k+\frac{n}{2}-1$. Thus,

(A215) $$\begin{array}{ccc} |\frac{{\left(\frac{\tau }{2}\right)}^{k}}{k!\;\Gamma \left(k+\frac{n}{2}\right)}\;{\left(\frac{u}{2}\right)}^{k+\frac{n}{2}-1}\;e^{-\frac{u}{2}}| & \leq & \frac{{\left(\frac{\tau }{2}\right)}^{k}}{k!\;\Gamma \left(k+\frac{n}{2}\right)}\;{\left(k+\frac{n}{2}-1\right)}^{k+\frac{n}{2}-1}\;e^{-\left(k+\frac{n}{2}-1\right)} \end{array}$$

(A216) $$\begin{array}{ccc} & = & \frac{{\left(\frac{\tau }{2}\right)}^{k}\;e^{\left(k+\frac{n}{2}-1\right)\ln\left(k+\frac{n}{2}-1\right)-\left(k+\frac{n}{2}-1\right)}}{k!\;\Gamma \left(k+\frac{n}{2}\right)}≜M_{k}. \end{array}$$

Next, we verify that

(A217) $$\begin{array}{ccc} \lim_{k\to \infty }\frac{M_{k+1}}{M_{k}} & = & \lim_{k\to \infty }\frac{{\left(\frac{\tau }{2}\right)}^{k+1}\;e^{\left(k+\frac{n}{2}\right)\ln\left(k+\frac{n}{2}\right)-\left(k+\frac{n}{2}\right)}}{\left(k+1\right)!\;\Gamma \left(k+\frac{n}{2}+1\right)}\cdot \frac{k!\;\Gamma \left(k+\frac{n}{2}\right)}{{\left(\frac{\tau }{2}\right)}^{k}\;e^{\left(k+\frac{n}{2}-1\right)\ln\left(k+\frac{n}{2}-1\right)-\left(k+\frac{n}{2}-1\right)}} \end{array}$$

(A218) $$\begin{array}{ccc} & = & \lim_{k\to \infty }\frac{\frac{\tau }{2}\;e^{\left(k+\frac{n}{2}\right)\ln\left(k+\frac{n}{2}\right)-\left(k+\frac{n}{2}-1\right)\ln\left(k+\frac{n}{2}-1\right)-1}}{\left(k+1\right)\;\left(k+\frac{n}{2}\right)} \end{array}$$

(A219) $$\begin{array}{ccc} & = & \lim_{k\to \infty }\frac{\frac{\tau }{2}\left(k+\frac{n}{2}-1\right)\;e^{\left(k+\frac{n}{2}\right)\ln\left(1+\frac{1}{k+n/2-1}\right)-1}}{\left(k+1\right)\;\left(k+\frac{n}{2}\right)}=0, \end{array}$$

because

(A220) $$\begin{array}{c} \lim_{k\to \infty }\;e^{\left(k+\frac{n}{2}\right)\ln\left(1+\frac{1}{k+n/2-1}\right)-1}=1. \end{array}$$

Thus, we see that Weierstrass’ M-test is satisfied and that thus (A49) is uniformly converging for all u≥0.

### Appendix E.2. Uniform Convergence of *(A55)*

We note that for any 0≤ξ≤Ξ:

(A221) $$\begin{array}{ccc} |\;e^{-\xi }\frac{\xi^{k}}{k!}\sum_{j=1}^{\frac{n-1}{2}+k}\frac{1}{j-\frac{1}{2}}| & \leq & \frac{\Xi^{k}}{k!}\sum_{j=1}^{\frac{n-1}{2}+k}\frac{1}{j-\frac{1}{2}} \end{array}$$

(A222) $$\begin{array}{ccc} & = & \frac{\Xi^{k}}{k!}\left(2+\sum_{j=2}^{\frac{n-1}{2}+k}\frac{1}{j-\frac{1}{2}}\right) \end{array}$$

(A223) $$\begin{array}{ccc} & \leq & \frac{\Xi^{k}}{k!}\left(2+\sum_{j=2}^{\frac{n-1}{2}+k}\frac{1}{j-1}\right) \end{array}$$

(A224) $$\begin{array}{ccc} & = & \frac{\Xi^{k}}{k!}\left(2+\sum_{j=1}^{\frac{n-1}{2}+k-1}\underset{\leq \;1}{\underbrace{\frac{1}{j}}}\right) \end{array}$$

(A225) $$\begin{array}{ccc} & \leq & \frac{\Xi^{k}}{k!}\left(2+\frac{n-1}{2}+k-1\right) \end{array}$$

(A226) $$\begin{array}{ccc} & = & \frac{\Xi^{k}}{k!}\left(k+\frac{n}{2}+\frac{1}{2}\right)≜M_{k}. \end{array}$$

Since

(A227) $$\begin{array}{ccc} \lim_{k\to \infty }\frac{M_{k+1}}{M_{k}} & = & \lim_{k\to \infty }\frac{\Xi^{k+1}\left(k+\frac{n}{2}+\frac{3}{2}\right)}{(k+1)!}\cdot \frac{k!}{\Xi^{k}\left(k+\frac{n}{2}+\frac{1}{2}\right)} \end{array}$$

(A228) $$\begin{array}{ccc} & = & \lim_{k\to \infty }\frac{\Xi \left(k+\frac{n}{2}+\frac{3}{2}\right)}{\left(k+1\right)\left(k+\frac{n}{2}+\frac{1}{2}\right)}=0, \end{array}$$

we see that Weierstrass’ M-test is satisfied and that thus (A55) is uniformly converging for all finite ξ.
